# Neutrophils From Children With Systemic Juvenile Idiopathic Arthritis Exhibit Persistent Proinflammatory Activation Despite Long-Standing Clinically Inactive Disease

**DOI:** 10.3389/fimmu.2018.02995

**Published:** 2018-12-18

**Authors:** Rachel A. Brown, Maggie Henderlight, Thuy Do, Shima Yasin, Alexei A. Grom, Monica DeLay, Sherry Thornton, Grant S. Schulert

**Affiliations:** ^1^Division of Rheumatology, Cincinnati Children's Hospital Medical Center, Cincinnati, OH, United States; ^2^Department of Pediatrics, University of Cincinnati College of Medicine, Cincinnati, OH, United States

**Keywords:** macrophage activation syndrome, S100, neutrophil subsets, gene expression, systemic JIA, autoinflammation

## Abstract

**Background:** Systemic juvenile idiopathic arthritis (SJIA) is a chronic childhood arthropathy with features of autoinflammation. Early inflammatory SJIA is associated with expansion and activation of neutrophils with a sepsis-like phenotype, but neutrophil phenotypes present in longstanding and clinically inactive disease (CID) are unknown. The objective of this study was to examine activated neutrophil subsets, S100 alarmin release, and gene expression signatures in children with a spectrum of SJIA disease activity.

**Methods:** Highly-purified neutrophils were isolated using a two-step procedure of density-gradient centrifugation followed by magnetic-bead based negative selection prior to flow cytometry or cell culture to quantify S100 protein release. Whole transcriptome gene expression profiles were compared in neutrophils from children with both active SJIA and CID.

**Results:** Patients with SJIA and active systemic features demonstrated a higher proportion of CD16^+^CD62L^lo^ neutrophil population compared to controls. This neutrophil subset was not seen in patients with CID or patients with active arthritis not exhibiting systemic features. Using imaging flow cytometry, CD16^+^CD62L^lo^ neutrophils from patients with active SJIA and features of macrophage activation syndrome (MAS) had increased nuclear hypersegmentation compared to CD16^+^CD62L^+^ neutrophils. Serum levels of S100A8/A9 and S100A12 were strongly correlated with peripheral blood neutrophil counts. Neutrophils from active SJIA patients did not show enhanced resting S100 protein release; however, regardless of disease activity, neutrophils from SJIA patients did show enhanced S100A8/A9 release upon PMA stimulation compared to control neutrophils. Furthermore, whole transcriptome analysis of highly purified neutrophils from children with active SJIA identified 214 differentially expressed genes (DEG) compared to neutrophils from healthy controls. The most significantly upregulated gene pathway was Immune System Process, including *AIM2, IL18RAP*, and *NLRC4*. Interestingly, this gene set showed intermediate levels of expression in neutrophils from patients with long-standing CID yet persistent serum IL-18 elevation. Indeed, all patient samples regardless of disease activity demonstrated elevated inflammatory gene expression, including inflammasome components and *S100A8*.

**Conclusion:** We identify features of neutrophil activation in SJIA patients with both active disease and CID, including a proinflammatory gene expression signature, reflecting persistent innate immune activation. Taken together, these studies expand understanding of neutrophil function in chronic autoinflammatory disorders such as SJIA.

## Introduction

Systemic juvenile idiopathic arthritis (SJIA) is a severe and distinctive subtype of juvenile idiopathic arthritis (JIA). Along with arthropathy, SJIA is characterized by quotidian fevers, evanescent rash, adenopathy, hepatomegaly, and serositis (1). Children with SJIA are also at risk for life-threating complications including macrophage activation syndrome (MAS) and severe lung disease (2, 3). The pathogenesis of SJIA is incompletely understood; however, it has many shared features with the monogenic autoinflammatory disorders. In contrast to autoimmune diseases, autoinflammatory disorders typically lack autoreactive lymphocytes or high-titer autoantibodies, and are instead defined by excessive and uncontrolled activation of innate immunity (4). In support of this, SJIA is characterized by increased circulating innate immune effectors, upregulation of monocyte/macrophage differentiation genes, as well as high levels of monocyte-derived proinflammatory cytokines including IL-1, IL-6, and IL-18 (5–12). Children with SJIA also typically have excellent clinical response to biologic therapy targeting IL-1 and IL-6 (13).

Neutrophils are the most numerous innate immune effectors in the circulation, and have key roles in both host defense and autoinflammation. Neutrophils ingest particulate material through phagocytosis, and kill microbes through overlapping oxidative and non-oxidative mechanisms (14). Neutrophils are also key sources of proinflammatory mediators, including the alarmin proteins S100A8/A9 (calprotectin) and S100A12 (calgranulin C), which amplify innate immune signaling (15). During severe systemic inflammation, neutrophils become rapidly activated, leading to reactive oxygen species (ROS) production, release of neutrophil extracellular traps (NETs), and changes in gene expression profiles (16). While neutrophils have classically been considered terminally differentiated cells with homogenous functions, increasing evidence suggests that functional neutrophil subsets emerge during states of systemic inflammation. This includes both immature “banded” neutrophils as well as mature neutrophils with immunomodulatory properties (16, 17). These later cells, sometimes termed “suppressive neutrophils,” are defined as CD16^+^CD62L^dim^, can display nuclear hypersegmentation, have distinct transcriptomes (18), and suppress T cell responses through ROS and direct cell contact (18–20).

Neutrophils also appear to have key roles in the pathogenesis of autoinflammatory disorders such as SJIA. Circulating neutrophils are markedly increased in active SJIA (21), and neutrophil numbers are associated with IL-1 related gene expression profiles in whole blood (22). Neutrophil-derived mediators such as S100 proteins are markedly elevated in active SJIA (23). A recent comprehensive analysis of neutrophil phenotypes in primarily new-onset disease significantly advanced the understanding of these cells in SJIA (24). This work found that neutrophil numbers strongly correlate with inflammatory disease parameters, have a primed and sepsis-like phenotype, and that circulating counts rapidly normalize with successful IL-1 blockade. In many children however, SJIA has a chronic and sustained disease course, associated with persistently elevated serum IL-18 levels and epigenetic changes in monocytes (12, 25). In contrast, little is known regarding whether neutrophil phenotypic and functional abnormalities persist in chronic SJIA and/or clinically inactive disease (CID).

In this study, we examined neutrophil subsets, functional properties, and gene expression signatures in large cohort of children with SJIA, including new-onset disease, active disease (both systemic and/or arthritic features), and CID. We hypothesized that active SJIA is associated with significant, proinflammatory changes in neutrophil phenotypes. We also hypothesized that these changes persist in patients with longstanding inactive disease but with signs of persistent immune activation.

## Materials and Methods

### Patients

This study was approved by the Institutional Review Board of Cincinnati Children's Hospital Medical Center (IRB 2016-2234), and written informed consent was obtained from all adult patients and from the parents or legal guardians of enrolled children. Systemic juvenile idiopathic arthritis (SJIA) was diagnosed based on the International League of Associations for Rheumatology diagnostic criteria (26), though for several patients, samples were obtained and treatment initiated with disease duration <6 weeks, in agreement with the operational definition of SJIA as described (27). Patients were considered as having active SJIA if they had presence of any active arthritis; any systemic features including rash, fever, adenopathy, or hepatosplenomegaly; or elevated ESR or CRP. Patients were considered to have CID based on the Wallace criteria (28). MAS was diagnosed per the treating physician; however, all MAS episodes also satisfied the 2018 MAS Classification Criteria (29). Patients were enrolled and peripheral blood samples were collected during routine visits, and laboratory information was gathered from testing done during the routine clinical care. Serum was collected, alloquated and stored at −80°C until analyzed.

Control samples were recruited from children undergoing evaluation for joint pain at the pediatric rheumatology clinic at CCHMC but found to have non-inflammatory conditions, as well as healthy young adult donors (ages 18–30) through the Cell Processing and Manipulation Core at CCHMC.

### Neutrophil Isolation and Purification

Neutrophils were isolated from fresh whole blood collected in ACD solution A vacutainer (Becton Dickinson, Franklin Lakes, NJ) tubes. Briefly, neutrophils were isolated through density gradient centrifugation as described (30). Cells were then further purified using magnetic-bead based negative selection with the MACSexpress Human Whole Blood Neutrophil Isolation kit (Miltenyi Biotec, Germany), followed by hypotonic lysis of remaining red blood cells. Neutrophil purity was assessed by flow cytometry (see below) and was typically >98%.

### Antibodies and Flow Cytometry

All antibodies were from BD Biosciences. Antibodies used for these studies were Pacific Blue conjugated anti-human CD14 (clone M5E2), FITC conjugated anti-human CD15 (clone MMA), BV711 or PE conjugated anti-human CD16 (clone 3G5), APC, or AF647 conjugated anti-human CD62L (clone DREG56), BV421 conjugated anti-human CD193 (clone 5E8), and PE conjugated anti-human Siglec8 (clone 7C9). Approximately 1 million neutrophils were stained for surface markers for 30 min at 4°C. For imaging cytometry experiments, cells were then stained with 300nM DAPI (Thermofisher, Waltham, MA) for 10 min. The cells were then washed with FACS buffer (PBS supplemented with 1% fetal calf serum) prior to filtering and transferred to 12 × 75 mm polystyrene tubes. Cells were acquired using a BD LSR Fortessa analytical cytometer. Data was analyzed by FACSDiva and FlowJo software.

Imaging flow cytometry was performed on an ImageStreamX (EMD Millipore) two camera system equipped with 405, 488, 642, and 785 nm lasers. Cells were imaged using the 60 × objective with the 785 nm laser turned off. Laser powers were set to optimize fluorescence detection with 405 nm set to 20 mW, 288 nm set to 20 mW, and the 642 nm set to 50 mW. Classifiers were set on brightfield to eliminate debris and on fluorescence channels to eliminate saturated images. Focused images of single cells were analyzed using the lobe count feature on the morphology mask of the DAPI signal to compare nuclear segmentation of CD16+ cells with either high or low expression of CD62L.

### Neutrophil Culture and S100 Alarmin Release

To quantify release of S100 alarmin proteins, 1 × 10^6^ isolated purified neutrophils were incubated in RPMI and either left untreated or stimulated with phorbol myristate acetate (PMA) for 4 h. Subsequently, culture supernatants were collected, centrifuged to remove cells, and stored at −80°C. S100A8/A9 was determined using specific ELISA kit obtained from ALPCO (Salem, NH), and serum S100A12 levels using specific ELISA kits obtained from MBL (Woburn, MA).

### Whole Transcriptome Analysis

Total RNA from purified neutrophils was extracted using the MagMax −96 Total RNA Isolation Kit (Life Technologies), and quantified via Qubit RNA HS Assay Kit (Life Technologies). Using the SuperScript VILO cDNA Synthesis Kit (Life Technologies), 10 ng of RNA was reverse transcribed to make cDNA. The Ion AmpliSeq Library Kit Plus (Life Technologies) and the Ion AmpliSeq Transcriptome Human Gene Expression Core Panel was used to amplify target genes per manufacturer's directions. Each amplicon was then barcoded with the Ion Express Barcode Adapter (Life Technologies), and purified through Agencourt AMPure XP Beads (Beckman Coulter) and freshly prepared 70% ethanol. Finally, libraries were analyzed with High Sensitivity NGS Fragment Analysis Kit (AATI). All libraries were peaked around 200 bp.

Before sequencing, the concentration of the library was determined via Qubit dsDNA HS kit (Life Technologies) and diluted with nuclease-free water to 100 pM. Using the Ion 540 Kit-OT2 along with the Ion OneTouch 2 Instrument, the library was amplified on the Ion Spheres Particles (ISP) through emulsion PCR. Then, template-positive ISPs was recovered, and their quality was assessed through the Ion Sphere Quality Control Kit and the Qubit 2.0 Fluorometer. The ISPs were then enriched with Dynabead MyOne Streptavidin C1 Beads to select the clonally amplified DNA. Sequencing primers were annealed to the enriched ISPs and loaded on the Ion 540 Chip along with the Ion S5 Sequencing Polymerase. The loaded chip was run on the Ion S5 sequencer, and the amplicon regions were mapped with hg19_AmpliSeq_Transcriptome_ERCC_V1 reference from Ion Community.

After reads were mapped they were converted into reads per kilobase of transcript per million mapped reads (RPKM). Differentially expressed genes (DEG) (fold change >2, *p* < 0.05) were determined using AltAnalyze (31). This package was also utilized to perform principle component analysis, hierarchical clustering of DEGs, and pathway analysis for significantly enriched gene ontology pathways and transcription factor targets. The gene expression datasets for this study can be found in Gene Expression Omnibus (GSE122552).

## Results

### Mature CD16^+^CD62L^dim^ Neutrophils in Systemically Active SJIA

**Figure 1:**
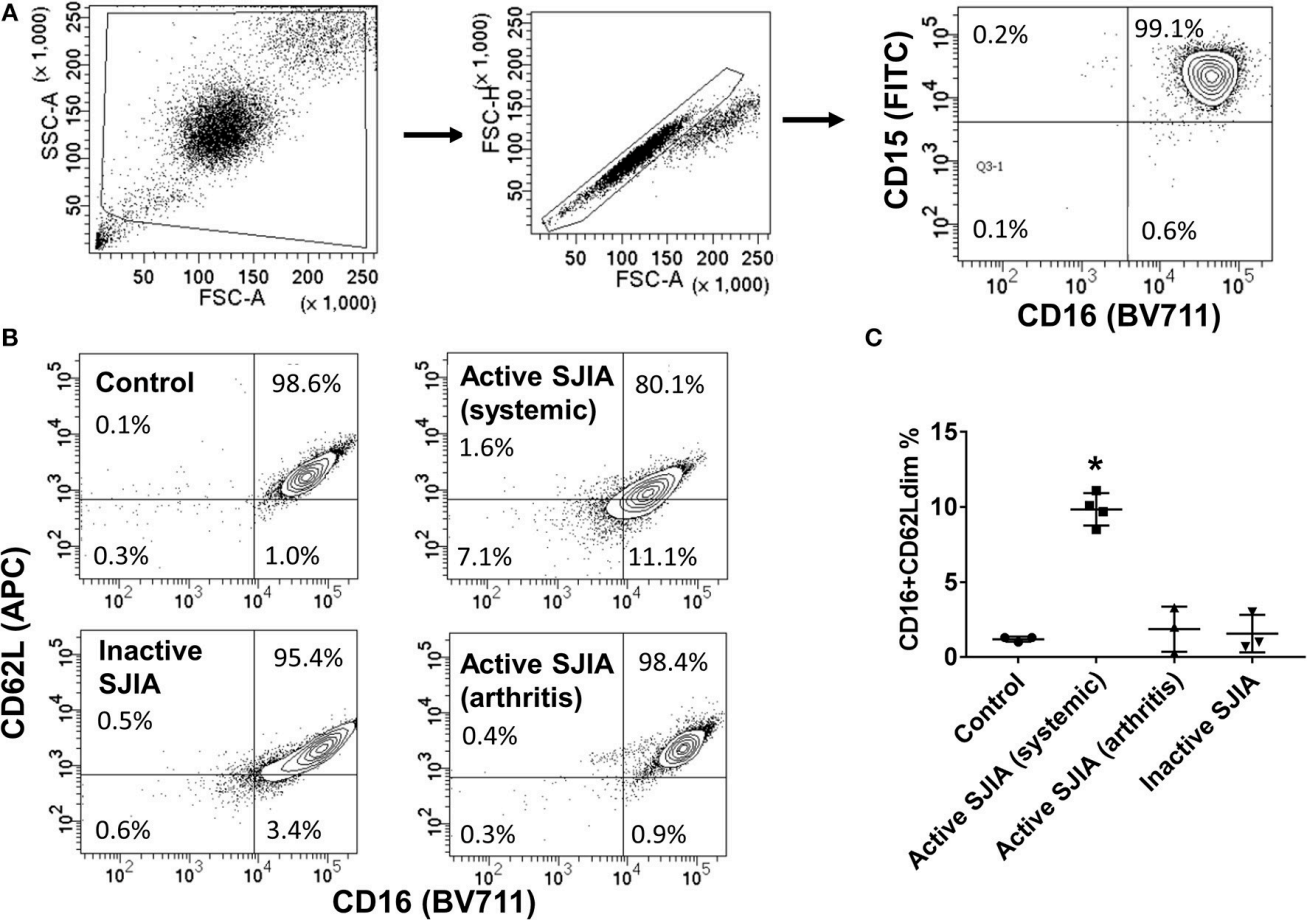
Flow cytometry analysis of highly purified neutrophil populations. Neutrophils were stained with FITC-conjugated anti-CD15, BV711-conjugated anti-CD16, and APC-conjugated anti-CD62L before being analyzed. **(A)** Cells were gated for live cells by FSC/SSC, then for doublet discrimination, then to identify percentage of CD15+CD16+ neutrophils. **(B,C)** Neutrophils were analyzed for relative percentage of mature CD16^+^CD62L^dim^ cells. Gates were set using healthy controls to define CD62L thresholds. Data are representative of control neutrophils, neutrophils from patients with SJIA and active systemic features, active arthritis only, and inactive SJIA. Pooled data are shown in **(C)**. ^*^p = 0.001 vs. control as determined by ANOVA with Dunnett's multiple comparisons test.

Circulating neutrophil counts are markedly elevated in patients with active SJIA, and recent work has described a sepsis-like phenotype during the early inflammatory phase at disease onset (24). To further characterize functional neutrophil properties throughout SJIA disease course, we utilized a two-step procedure to obtain highly purified (>98%) and untouched cell populations (Figure 1A). First, granulocytes were separated through density gradient centrifugation (30). Following this, a magnetic-bead based negative selection step further purified the neutrophil suspension. Of note, this procedure produced populations with minimal (<1%) contamination with CD14+ monocytes, the higher RNA content of which can alter interpretation of transcriptional profiles (32), or from CD193+Siglec8+ eosinophils (data not shown).

Purified peripheral blood neutrophils were obtained from a large cohort of patients with SJIA including various states of disease activity including new-onset disease, active disease (both systemic and/or arthritic features) and CID, as well as both pediatric and adult healthy controls (Table 1). While circulating mature neutrophils had generally been considered to be a homogenous cell population, recent work has identified functional neutrophil subsets emerging in states of systemic inflammation (19). These include neutrophils described as CD16^+^CD62L^dim^, and capable of suppressing T cell proliferation through integrins and ROS production (17). We examined purified neutrophils from SJIA patients with both active and inactive disease for presence of this CD16^+^CD62L^dim^ subset. Approximately 10% of neutrophils from children with SJIA and active systemic features were CD16^+^CD62L^dim^, which was significantly higher than that seen with control neutrophils (Figures 1B,C). Patients with active SJIA also manifested lower neutrophil CD16 signal, possibly reflecting either an increase in both immature banded neutrophils (CD16^dim^ CD62L^+^) and apoptotic neutrophils (CD16^dim^CD62L^dim^) and/or shedding of CD16 by sustained neutrophil activation (33). In contrast, patients with either CID or active SJIA with only arthritis and no systemic features did not show increases in CD16^+^CD62L^dim^ (Figures 1B,C). These findings suggest that mature CD16^+^CD62L^dim^ neutrophils are specific to the systemic inflammatory phase of SJIA.

**Table 1:** Clinical and laboratory characteristics of patients enrolled for neutrophil collection.

	**Active SJIA (*n* = 23)**	**Inactive SJIA (*n* = 22)**
Age, median	8 (5–14)	11 (6.75–16)
Sex	11F, 12M	13F, 9M
Ferritin (ng/mL)	559.5 (65.6–5379)	23.5 (17.25–33.5)
CRP (mg/dL)	5.04 (0.22–9.88)	<0.29 (<0.29 to <0.29)
ESR (mm/hr)	59 (15–76)	5 (2–9)
IL-18 (pg/mL)	19852 (3632–111678)	956 (321–2697)
S100A8/A9 (ng/mL)	6869 (442.5–33116)	1615 (680–3431)
S100A12 (ng/mL)	220 (56–605.8)	76 (55–182)
Fever	34.8% (8/23)	N/A
Arthritis	70.0% (16/23)	N/A
Systemic features	34.8% (8/23)	N/A
Elevated ESR/CRP	82.6% (19/23)	N/A
New-onset SJIA	17.4% (4/23)	N/A
MAS or subclinical MAS	21.7% (5/23)	N/A
Time in CID, median	N/A	8 months (5.75–18.5)
History of MAS	60.9% (14/23)	31.8% (7/22)
History of chronic lung disease	17.4% (4/23)	4.5% (1/22)

*IL-18 standard ranges 89–540 pg/mL. S100A8/A9 standard ranges 716–3004 ng/mL. S100A12 standard ranges 32–385 ng/mL*.

**Figure 2:**
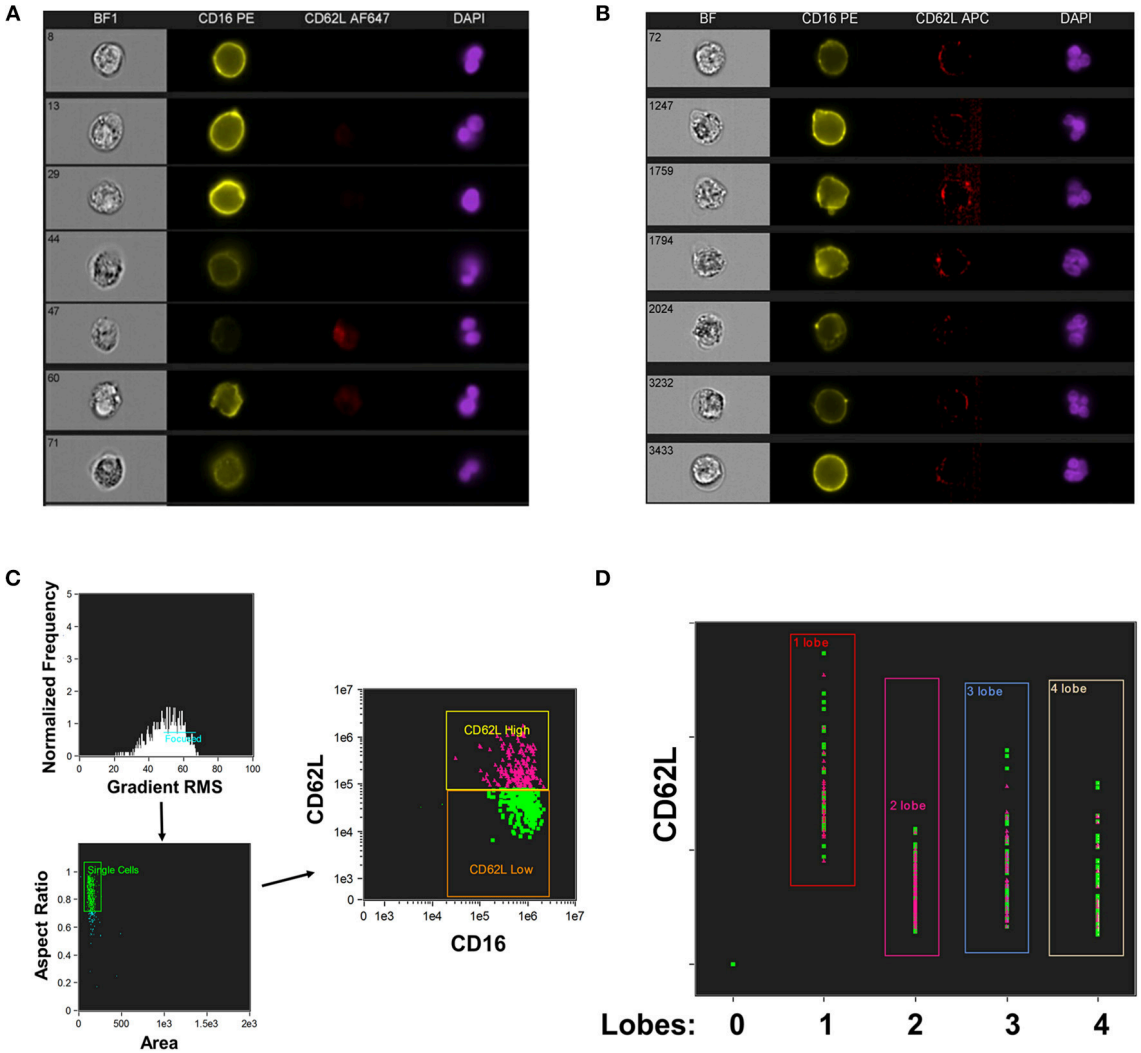
Nuclear segmentation in neutrophils from active SJIA. Highly purified neutrophils were stained with PE-conjugated anti-CD16, APC- or AF647-conjugated anti-CD62L, and DAPI nuclear stain before analyzing with the Imagestream imaging cytometer. **(A,B)** representative images of neutrophils scored as having 2 lobes **(A)** and ≥4 nuclear lobes **(B)**. **(C,D)** Analyzed neutrophils from a patient with active SJIA and features of subclinical MAS. **(C)** Cells were gated for focused cells, single cells, and finally for CD16^+^CD62L^+^ (pink) and CD16^+^CD62L^dim^ (green) neutrophils. **(D)** Distribution of CD16^+^CD62L^+^ (pink) and CD16^+^CD62L^dim^ (green) neutrophils by number of identified nuclear lobes.

CD16^+^CD62L^dim^ neutrophils have also been reported to have a hypersegmented nuclear appearance, both when associated with systemic LPS administration (19) and with *Helicobacter pylori* infection (34). To quantify this process, we utilized imaging cytometry to determine the number of nuclear lobes present in CD16^+^CD62L^+^ and CD16^+^CD62L^dim^ neutrophils. Using DAPI nuclear staining and spot counting, we could discriminate subpopulations of neutrophils with bi-lobed nuclei (Figure 2A) from those with hypersegmented nuclei (>4 nuclear lobes; Figure 2B). Through this imaging cytometry approach, we found that ~10% of neutrophils in all samples had ≥4 nuclear lobes. However, in SJIA patients with systemically active disease and features of early/subclinical MAS, substantially more hypersegmented cells were visualized, particularly amongst the CD16^+^CD62L^dim^ population compared to CD16^+^CD62L^+^ cells (22.9% vs. 11.3%; Figures 2C,D). Together, this demonstrates that systemically active SJIA patients have circulating, mature CD16^+^CD62L^dim^ neutrophils.

### Neutrophils From Both Active and Inactive SJIA Have Enhanced S100A8/A9 Release Capacity

**Figure 3:**
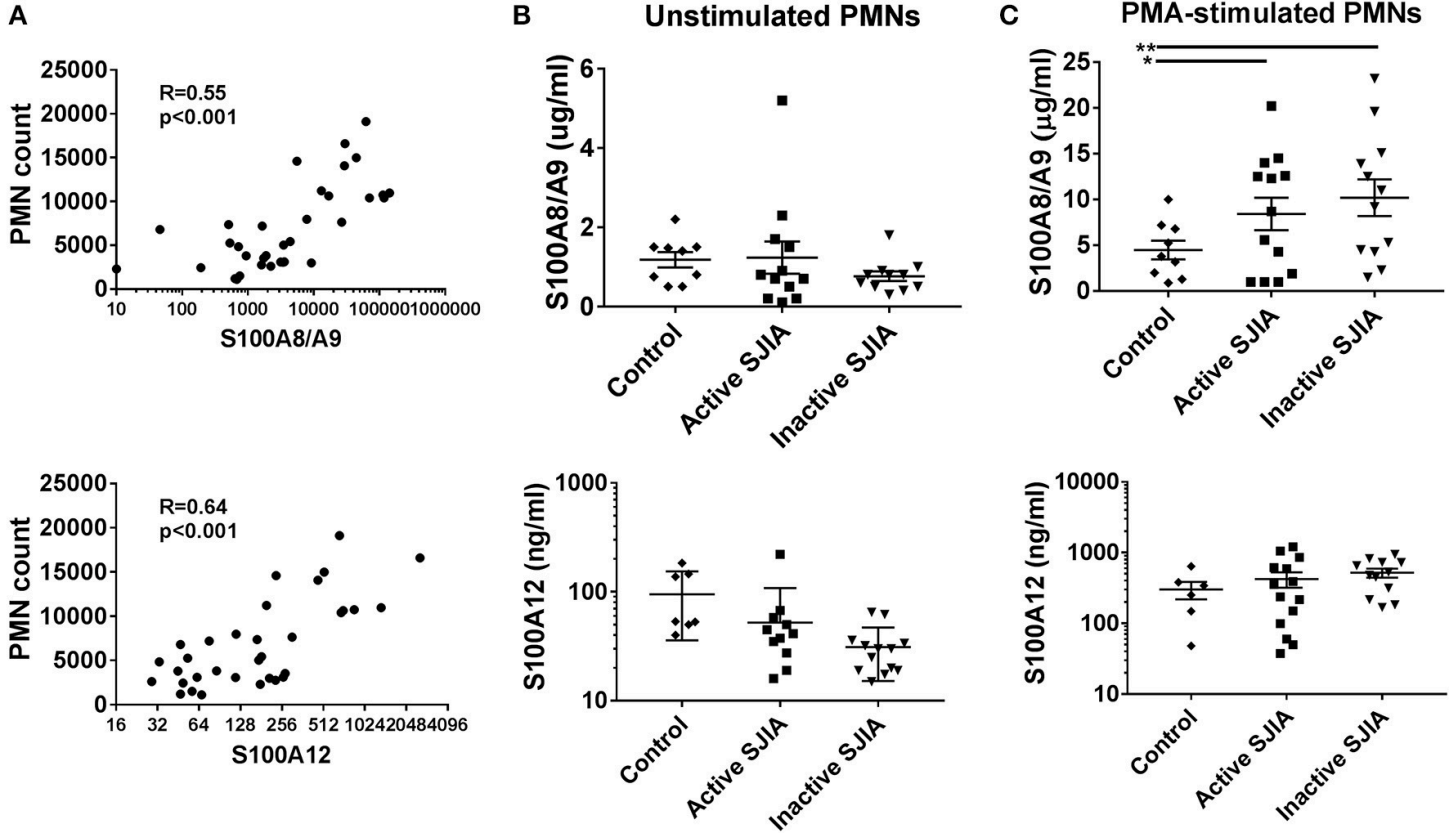
S100 alarmin proteins release by neutrophils in SJIA. **(A)** Correlation between circulating neutrophil count and serum S100A8/A9 (top) and S100A12 (bottom) in patients with SJIA. **(B,C)** S100 alarmin protein release from cultured neutrophils either at rest **(B)** or after PMA stimulation **(C)**. Top panels represent S100A8/A9, bottom panels S100A12. Error bars represent mean ± SEM. ^*^p < 0.05, ^**^P < 0.01.

S100 alarmin proteins, including S100A8/A9 (calprotectin) and S100A12 (calgranulin C), are host derived proinflammatory mediators that amplify innate immune responses by signaling through pattern recognition receptors (PRR) including TLR4 (15). These proteins are believed to be produced primarily by activated phagocytes, and present at very high levels in systemic inflammation as seen in SJIA (23). First, we confirmed that patients with active SJIA had significantly higher serum levels of S100A8/A9 and S100A12 than those with inactive disease (Table 1). We also confirmed prior work that peripheral neutrophil counts strongly and significantly correlate with serum S100A8/A9 (*R* = 0.55, *p* < 0.001) and S100A12 (*R* = 0.64, *p* < 0.001) levels (Figure 3A). In contrast, there was no significant correlation between the absolute lymphocyte count and S100A8/A9 levels (*R* = −0.08 and 0.07, respectively).

Recent work has shown that in the autoinflammatory disease familial Mediterranean fever (FMF), during active disease peripheral neutrophils constitutively and spontaneously release high levels of S100A12 (35). In order to quantify S100 protein release, 1 × 10^6^ highly purified neutrophils were placed in tissue culture and incubated for 4 h. After incubation, cell-free supernatants were collected to determine S100A8/A9 and S100A12 release. Interestingly, there was no significant difference in levels of S100 alarmin proteins released by unstimulated neutrophils during either active or inactive SJIA (Figure 3B). We also determined the amount of S100 proteins that could be released by neutrophils upon stimulating with PMA to induce activation and degranulation. Compared to control neutrophils, cells from patient with both active and inactive SJIA released significantly more S100A8/A9 upon PMA stimulation (Figure 3C). There was no significant difference in amount of S100A12 released by neutrophils from either active or inactive disease. Taken together, these findings suggest that even during CID, neutrophils have increased capacity for S100A8/A9 alarmin release upon cell activation.

### Proinflammatory Neutrophils Gene Expression Signatures in Both Active and Inactive SJIA

**Figure 4:**
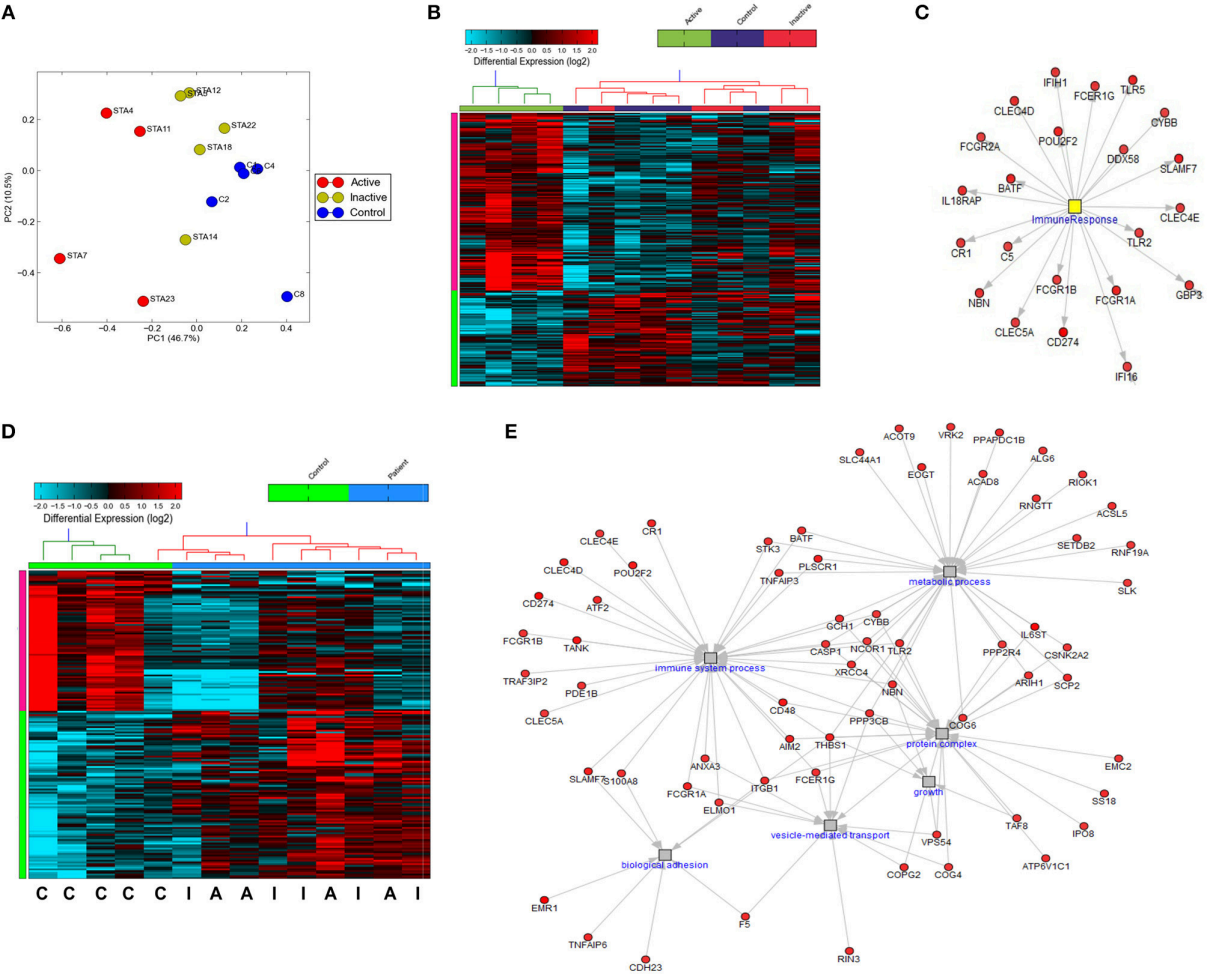
Gene expression profiling of highly purified neutrophils from patients with SJIA. **(A)** Principle component analysis of differentially expressed genes (DEG) between active SJIA and control neutrophils. **(B)** Hierarchical clustering of DEGs between active SJIA and control neutrophils (fold change >2.0, *p* < 0.05). **(C)** Network analysis of immune response transcription factor cluster genes upregulated in active SJIA neutrophils. **(D)** Hierarchical clustering of DEGs between all SJIA patients and control neutrophils (fold change >2.0, *p* < 0.05). **(E)** Network analysis of genes in significantly enriched GO pathways upregulated in all SJIA patients.

While previous work has described gene expression profiling of neutrophils from small cohorts of children with active SJIA (24, 36), little is known regarding these signatures in longstanding and inactive disease. We utilized the Ampliseq Transcriptome platform to determine gene expression profiles from highly purified neutrophils. Ampliseq Transcriptome is an amplicon-based gene expression system with near whole transcriptome coverage (>20,000 coding genes) and high correlation to microarray and RNA-sequencing (37). Gene expression profiling was performed on 14 neutrophil samples: (1) 4 from patients with systemically active SJIA but without MAS features, (2) 5 from patients with CID on medication, and (3) 5 healthy controls (Supplemental Table 1). Of note, the included patients with CID had longstanding remission on medication (6–48 months) but had persistently elevated serum IL-18 levels at least twice upper limit of normal (1163–8729 ng/ml). Principal component analysis showed clear distinction between transcriptomes of active SJIA neutrophils from control neutrophils, with inactive disease samples showing intermediate changes as compared to neutrophils from controls (Figure 4A). Using cut-offs of >2.0 fold change and *p* < 0.05, 139 genes were significantly upregulated and 75 genes significantly downregulated in neutrophils from active SJIA compared to controls. Hierarchical clustering based on these DEG is shown in Figure 4B; full list of DEG is shown in Supplemental Table 2. These genes included cell surface and PRR such as Fc-gamma receptor genes*, CR1, TLR2*, and *TLR5*; cytosolic PRR including *AIM2, NLRC4*, and *DDX58*; and *IL18RAP*.

To identify functional pathways of DEG in active SJIA neutrophils, Gene Ontology (GO) analysis was performed using AltAnalyze. The most significantly enriched GO pathways among upregulated DEG are shown in Table 2. The top GO pathway was Immune System Process (adjusted *p* = 3.44 × 10^−16^), with other immune/inflammatory pathways also highly enriched. The specific DEG contributing to this enriched pathway are shown in Table 3. Among downregulated DEG, no GO pathways had an adjusted *p* < 0.05; the most significantly enriched pathway was negative regulation of cellular metabolism (raw *p* = 0.005; z-score 3.11). When examining upregulated DEG for enriched transcription factor regulatory circuits, the most enriched circuit was immune response genes (Figure 4C). Specific transcription factor binding sites among upregulated genes included ISGF-3 (*p* = 0.006), STAT3 (*p* = 0.02), and Elf-1 (*p* = 0.04). Of note, ISGF-3 can be activated by type I interferon signaling (38, 39). Multiple serum cytokine studies have failed to identify type I or type II interferon signatures in the peripheral blood of SJIA patients with active disease (40, 41). Similarly, while we did find a significant enrichment in “regulation of type I interferon production” GO pathway (*p* = 0.003), this was driven by a small number of genes with pleotropic activation (*DDX58, IFI16, IFIH1, NFKBIA*, and *TLR2*), none of which were found to be highly specific for type I interferon responses (42).

**Table 2:** Gene ontology pathways of upregulated genes in neutrophils from children with active SJIA.

**Gene ontology pathway**	**Number changed**	**Number measured**	**Z score**	**Fisher exact test *P***	**Adjusted *P***
Immune system process (GO:0002376)	51	2091	10.88	3.78E-18	3.44E-16
Defense response (GO:0006952)	39	1284	11.13	8.98E-17	7.06E-13
Response to wounding (GO:0009611)	24	577	10.76	2.64E-13	1.04E-09
IgG binding (GO:0019864)	5	11	18.66	4.43E-09	5.80E-06
Response to other organism (GO:0051707)	16	460	7.73	4.21E-08	3.15E-05
Cell activation (GO:0001775)	18	594	7.42	4.62E-08	3.23E-05
Negative regulation of apoptosis (GO:0043066)	17	665	6.31	1.21E-06	0.000613
Response to lipopolysaccharide (GO:0032496)	10	229	7.12	2.22E-06	0.000965
External side of plasma membrane (GO:0009897)	9	196	6.98	4.84E-06	0.001901
Positive regulation of sequence-specific DNA binding transcription factor activity (GO:0051091)	9	199	6.91	5.47E-06	0.002048

**Table 3:** Immune System Process Gene Ontology pathway significantly upregulated genes in neutrophils from active SJIA.

**Gene symbol**	**Description**	**Active vs. control (log-fold change)**	**Active vs. control (*P*-value)**
NLRC4	NLR family, CARD domain containing 4	1.422914671	0.019850074
PLSCR1	Phospholipid scramblase 1	1.836792175	0.004685105
CR1	Complement component (3b/4b) receptor 1	1.465308433	0.036471508
CLEC5A	C-type lectin domain family 5, member A	1.306982102	0.008155234
POU2F2	POU class 2 homeobox 2	2.273993247	0.001974801
FCAR	Fc fragment of IgA, receptor for	1.039351747	0.042448537
THBS1	Thrombospondin 1	1.730758578	0.031004871
CLEC4E	C-type lectin domain family 4, member E	1.196901191	0.026681298
BATF	Basic leucine zipper transcription factor, ATF-like	2.098660806	0.001496821
TLR2	Toll-like receptor 2	1.310788972	0.047550579
SERPINB9	Serpin peptidase inhibitor, clade b (ovalbumin), member 9	2.279513514	0.014669102
CYBB	Cytochrome b-245, beta polypeptide	1.251099322	0.026243187
FCGR1B	Fc fragment of IgG, high affinity Ib, receptor (CD64)	1.364191125	0.010664911
CTSH	cathepsin H	1.471367492	0.036661403
PDE1B	Phosphodiesterase 1B, calmodulin-dependent	1.059161606	0.018171438
CX3CR1	Chemokine (C-X3-C motif) receptor 1	1.137782624	0.007845546
FCGR1A	Fc fragment of IgG, high affinity Ia, receptor (CD64)	2.18776973	0.019294383
SLAMF7	SLAM family member 7	2.336817458	0.013387457
IFIH1	Interferon induced with helicase C domain 1	1.373101981	0.040081759
MEF2A	Myocyte enhancer factor 2A	1.328120472	0.019727678
LGALS8	Lectin, galactoside-binding, soluble, 8	1.043496856	0.043430423
CD274	CD274 molecule	3.231874521	0.000717605
ANXA1	Annexin A1	1.314782826	0.006487109
GBP3	Guanylate binding protein 3	1.634893254	0.038159331
VNN1	Vanin 1	2.111799896	0.006807828
DDX58	DEAD (Asp-Glu-Ala-Asp) box polypeptide 58	1.159652732	0.015990704
NBN	Nibrin	1.760176462	0.041724081
NFKBIA	Nuclear factor of kappa light polypeptide gene enhancer in B-cells inhibitor, alpha	1.293830277	0.006803898
AIM2	Absent in melanoma 2	2.083271913	0.003477437
FCAR	Fc fragment of IgA, receptor for	1.039351747	0.042448537
IFI16	Interferon, gamma-inducible protein 16	1.291412088	0.001107247
FCGR2A	Fc fragment of IgG, low affinity IIa, receptor (CD32)	1.156808218	0.021307235
TANK	TRAF family member-associated NFKB activator	2.495474762	0.030111499
CLEC5A	C-type lectin domain family 5, member A	1.306982102	0.008155234
FCER1G	Fc fragment of IgE, high affinity I, receptor for; gamma polypeptide	1.452965375	0.046330661
CLEC4D	C-type lectin domain family 4, member D	1.755561907	0.005050456
TNFAIP3	Tumor necrosis factor, alpha-induced protein 3	2.137983366	0.003672704
FCAR	Fc fragment of IgA, receptor for	1.039351747	0.042448537
CYBB	Cytochrome b-245, beta polypeptide	1.251099322	0.026243187
LNPEP	leucyl/cystinyl aminopeptidase	1.326215551	0.02050466
FCAR	Fc fragment of IgA, receptor for	1.039351747	0.042448537
C5	Complement component 5	1.180276747	0.019419926
EDN1	Endothelin 1	1.357923043	0.021389531
TLR5	Toll-like receptor 5	2.129276632	0.014710728
IL18RAP	Interleukin 18 receptor accessory protein	1.737091117	0.006320721
CD48	CD48 molecule	2.1469991	0.000478521
DUSP3	Dual specificity phosphatase 3	1.034227595	0.040091537
SELP	Selectin P (granule membrane protein 140kDa, antigen CD62)	1.175328323	0.040344487
ANXA3	Annexin A3	1.526571135	0.001808271
TRIM22	Tripartite motif containing 22	1.324747508	0.005971802
FCGR1A	Fc fragment of IgG, high affinity Ia, receptor (CD64)	2.18776973	0.019294383
SMAD3	SMAD family member 3	1.765847458	0.015380056
FCGR1B	Fc fragment of IgG, high affinity Ib, receptor (CD64)	1.364191125	0.010664911
IRAK2	Interleukin-1 receptor-associated kinase 2	1.185197716	0.035499696

Interestingly, hierarchical clustering based on DEG from active SJIA neutrophils also largely differentiated neutrophils from patients with CID from controls (Figure 4B). Indeed, 4/5 inactive disease samples clustered together, with intermediate expression of upregulated DEG, while 4/5 control samples clustered separately with the lowest expression of this signature. To further investigate neutrophil gene expression changes in CID, we determined DEG between all SJIA patients (active and inactive disease) and controls. This revealed 97 significantly upregulated genes and 81 downregulated genes (fold change >2.0, *p* < 0.05; Supplemental Table 3). When this signature was used for hierarchical clustering, clear distinction was shown between patients and controls (Figure 4D). Neutrophils from SJIA patients clustered into two distinct groups; interestingly however, each contained samples from patients with both active and inactive disease. These DEG in all SJIA patient samples included *S100A8*, further supporting increased S100 release capacity as described above. GO analysis of upregulated DEG identified an overlapping network of pathways including immune system process (*p* = 1.28 × 10^−11^), vesicle-mediated transport (adjusted *p* = 8.23 × 10^−4^), protein complex (*p* = 0.02), and metabolic process (*p* = 0.04) (Figure 4E). Together, these findings suggest that in SJIA, neutrophils demonstrate a marked proinflammatory gene expression signature, and this can persist despite longstanding and clinically effective biologic treatment.

### Proinflammatory Neutrophil Gene Expression Signature During Inactive Disease With High and Normal Serum IL-18

**Figure 5:**
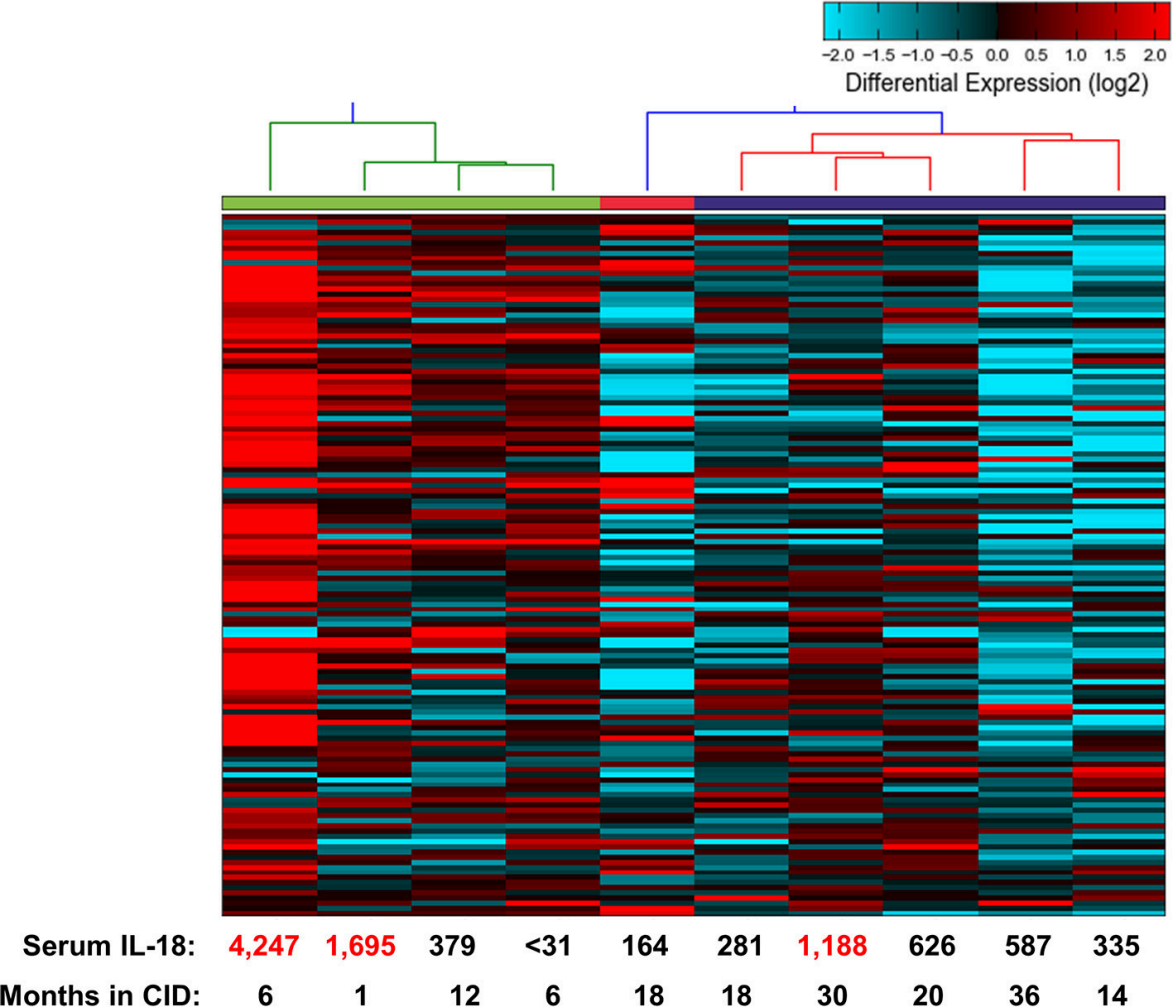
Proinflammatory gene expression signature in neutrophils from patients with CID. Unsupervised clustering performed using 139 upregulated DEGs identified as upregulated in neutrophils from active SJIA patients. Serum IL-18 levels at time of sample collection indicated on bottom. Numbers in red indicate values more than twice upper limit of normal.

The above findings are particularly intriguing given that the CID patients, while in longstanding remission on medication, had persistently elevated serum IL-18 levels. To examine this further, we determined gene expression signatures from a second cohort of patients with SJIA. RNA extracted from highly enriched neutrophils from 10 patients with inactive SJIA was sequenced using Ampliseq Transcriptome. These patients had all been in longstanding remission (6–36 months); however, only three patients had serum IL-18 levels greater than twice normal (1188–4247 ng/ml; Supplemental Table 4). These gene expression profiles were then utilized for unsupervised clustering using the upregulated DEG signature found above in active SJIA neutrophils. As shown in Figure 5, these samples showed a broad range of expression levels of this gene set. However, samples clustered into two primary groups, with 2/4 samples in the highest expression group from “high IL-18” patients and 5/6 samples in the lowest expression group from “low IL-18” patients. Samples also largely clustered based on time in CID, with samples <12 months in CID showing higher expression of this signature (Figure 5), possibly relating to the gradual decline of serum IL-18 over many months of treatment reported in SJIA (43). Together, these findings first confirms the persistence of proinflammatory gene expression signatures in neutrophils from patients with inactive SJIA, and second suggest an association between signature persistence and elevated serum IL-18 levels.

## Discussion

Neutrophils serve as key innate immune effector cells in systemic inflammation. In the early inflammatory phase of SJIA, neutrophils are markedly expanded and activated with a sepsis-like phenotype (24); however, cell properties in long-standing and CID are less defined. Here, we examined neutrophil subsets, S100 alarmin release, and gene expression profiles from a large cohort of SJIA patients across disease states. Taken together, this work has several important and novel findings. First, we identified hypersegmented, CD16^+^CD62L^dim^ suppressor neutrophils only in SJIA patients with active systemic disease, including features of MAS. Second, in contrast to FMF, neutrophils from SJIA patients did not constitutively release high levels of S100 proteins. Third, neutrophils from patients with both active and inactive SJIA had significantly increased capacity to release S100A8/A9 upon activation. Fourth, these neutrophils had a marked proinflammatory gene expression signature that is present in both active disease and longstanding CID on medication. Finally, proinflammatory gene expression profiles were associated with persistent serum IL-18 elevations. These findings expand our understanding of neutrophil phenotypes in SJIA, particularly during inactive disease.

The traditional view of neutrophils as short-lived cells with little capacity for phenotypic or transcriptional diversity has been challenged by the description of numerous functional neutrophil subsets (17). Among these are CD16^+^CD62L^dim^ cells which have been called “suppressor neutrophils” due to their capacity to inhibit T cell responses (19). CD16^+^CD62L^dim^ neutrophils have recently been shown to have distinct proteomic profiles, with an upregulation in pathways involved in adhesion and activation, response to stimuli, and immune processes, further supporting these cells representing a functional subset in systemic inflammation (44). Recent work has reported that ~10–20% of circulating neutrophils in active SJIA are CD16^+^CD62L^dim^ (24), similar to that observed here. We have confirmed and extended those observations to note that this population was only increased in patients with active systemic features (fever, rash, liver enlargement), and not in children who had only active arthritis. CD16^+^CD62L^dim^ neutrophils seen after experimental LPS challenge in human volunteers (19) or after infection by *H. pylori* (34) are reported to exhibit nuclear hypersegmentation. Here, we utilized imaging cytometry to quantify nuclear segmentation, and found that in patients with systemically active disease, more CD16^+^CD62L^dim^ neutrophils had ≥4 visualized lobes than CD16^+^CD62L^+^ cells, particularly in patients with features of MAS. This is unsurprising, given the emerging view of both severe sepsis and MAS as “cytokine storm syndromes” with similar immune dysregulation (45). The significance of nuclear hypersegmentation, and of CD16^+^CD62L^dim^ neutrophils more broadly in early inflammatory SJIA, remains to be determined. Of note, there is no direct evidence regarding the functional roles of neutrophils in SJIA pathogenesis, including whether CD16^+^CD62L^dim^ neutrophils have suppressive properties *in vivo*.

Phagocytes including neutrophils are key sources for S100 alarmin production during inflammation (15). S100A8/A9 and S100A12 levels are markedly elevated in active SJIA, and may serve as useful biomarkers to distinguish SJIA from other disorders (46–49). These levels also strongly correlate with circulating neutrophil counts in SJIA (24). Recently, Gohar et al. reported that in the monogenic autoinflammatory disorder FMF, unstimulated neutrophils from patients with *MEFV* mutations spontaneously secreted S100A12 (35). In contrast, we report here that neutrophils from children with active or inactive SJIA do not show spontaneous S100A8/A9 or S100A12 release. This likely relates to the multifactorial nature of SJIA vs. the specific role of pyrin dysfunction in FMF. In contrast, we found that when activated by PMA, neutrophils from SJIA patients secreted significantly more S100A8/A9, regardless of disease activity. It is unknown why S100A8/A9 release was comparable between neutrophils from patients with active and inactive disease despite transcriptional profiles suggesting an intermediate phenotype, but this suggests the need for further linkage between gene expression and functional/protein data. The clinical implications of enhanced S100A8/A9 release capacity are unclear; however, it suggests that there are persistent changes in neutrophil phenotypes even in longstanding CID. In support of this, ter Haar et al. reported extensive *ex vivo* analysis of neutrophils from patients with active SJIA, demonstrating increased surface expression of degranulation markers and a primed phenotype for response to N-formyl peptides (24). While most of these changes reverted in neutrophils from patients with inactive disease, some, such as surface CD35 expression, had an intermediate phenotype reflecting increased degranulation.

Although neutrophils have been historically considered to have limited capacity for gene expression, recent findings instead suggest that they can undergo functional transcriptional alterations including epigenetic modifications (50). Complicating this analysis is that neutrophils generally contain 10–20 fold less mRNA than PBMC (32), and thus even in 95% pure cell populations other cell types may be large contributors to the gene expression signature. Here, we utilized a two-step purification to collect an “untouched” >98% pure neutrophil populations, with <1% contamination from blood monocytes or eosinophils (Figure 1A). Detection of IL6 mRNA, typically absent from neutrophils, has also been suggested as a marker for cell contamination (32); in all samples we detected <1 copy *IL6* per million reads.

Compared to control neutrophils, cells from children with active SJIA displayed a proinflammatory gene expression signature, including upregulation in PRR, inflammasome components, and the IL-18 receptor component *IL18RAP*. Although this sequencing was performed on highly purified but unsorted neutrophils, these changes are likely not solely due to the presence of CD16^+^CD62L^dim^ neutrophils in active disease. Gene expression studies on sorted CD16^+^CD62L^dim^ neutrophils during human experimental sepsis found the most upregulated genes compared to CD16^+^CD62L^+^ cells were in signal transduction and regulation of apoptosis pathways (18), which were not highly represented in the present study. In addition, we found that the proinflammatory signature identified here was also present in neutrophils from children with longstanding clinically-inactive SJIA. This neutrophil gene expression signature was broadly similar to that found in sorted neutrophils from patients with early inflammatory SJIA (24), although that study did not examine cells from patients with CID. Another small study examining gene expression in neutrophils from SJIA patients related to tocilizumab treatment found primarily changes related to mitochondrial and oxidative stress genes (36). Interestingly this study did not find substantive changes in proinflammatory pathways pre- and post-treatment, supporting our findings of persistence of gene expression changes despite clinically effective treatment. Further work utilizing single-cell approaches for both gene expression and protein marker expression are needed to confirm and extend this work.

The changes in neutrophil gene expression profile reported here are particularly interesting in comparison to those reported in other inflammatory arthropathies affecting adults and children. Extensive work by Jarvis et al. has defined a proinflammatory phenotype of neutrophils from non-systemic, rheumatoid factor-negative polyarticular JIA (51–54). In this disorder however, neutrophils demonstrated upregulated gene clusters linked to IL-8 and IFNγ, which were not highly represented in the present study. Indeed, only 4/42 most highly upregulated genes in neutrophils from polyarticular JIA were identified in our patients with SJIA (54). On the other hand, Jarvis et al. did find that neutrophil alternations in polyarticular JIA persisted in disease remission, supporting a model of long-term cell alterations in JIA (53). Gene expression studies of neutrophils in adults with rheumatoid arthritis similarly identified IFN signaling as the most differentially regulated pathway, distinguishing patients with improved response to treatment (55). Findings in this variety of disorders also highlight the capacity of neutrophils for highly specific transcriptional responses in the setting of distinct pathological settings (51, 52).

Together, we report both functional and gene expression evidence of neutrophil alterations in SJIA patients with longstanding CID. This is in agreement with our previous data on monocytes, which found persistent epigenetic changes in children with inactive disease (25). Of note, more than half of patients with CID in the present cohort continued to have elevated serum IL-18 levels. There is significant evidence that IL-18 has pleotropic effects on neutrophil activation, including cytokine gene expression and release, degranulation, and priming of the oxidative burst (56–58). Indeed, we demonstrated that persistently high IL-18 levels were associated with proinflammatory neutrophil gene expression signatures in patients with CID. IL-18 is also reported to trigger further autocrine expression and secretion of this cytokine by neutrophils, potentially implicating neutrophils in a feed-forward process to perpetuate IL-18 production. As such, further studies are needed to define the role of neutrophil subpopulation activation in both early inflammatory SJIA and in persistence of chronic disease.

## Author Contributions

RB, AG, and GS designed the study. RB, MH, TD, SY, and GS collected samples, isolated cells, and performed flow cytometry and cell culture experiments. TD performed gene expression profiling experiments. MD and ST performed imaging cytometry. RB, ST, AG, and GS analyzed the data. All authors contributed to drafting of the work and approved the final manuscript.

### Conflict of Interest Statement

AG has served as a consultant for Jun and Novartis, and has received research support from NovImmune and AB2Bio. The remaining authors declare that the research was conducted in the absence of any commercial or financial relationships that could be construed as a potential conflict of interest.
