# Supplementary material for: Neutrophils From Children With Systemic Juvenile Idiopathic Arthritis Exhibit Persistent Proinflammatory Activation Despite Long-Standing Clinically Inactive Disease
Source: Front Immunol. 2018 Dec 18;9:2995. doi: 10.3389/fimmu.2018.02995 (PMC6305285; doi:10.3389/fimmu.2018.02995)
Supplement: Supplementary file 1 [file Table_1.DOCX]

Supplemental table 1: Patients in RNA-seq experiment 1

|  | 1 | 2 | 3 | 4 | 5 | 6 | 7 | 8 | 9 |
| --- | --- | --- | --- | --- | --- | --- | --- | --- | --- |
| Disease activity | Active | Active | Active | Active | CID | CID | CID | CID | CID |
| Age | 26 | 13 | 5 | 5 | 23 | 16 | 13 | 8 | 16 |
| Sex | M | M | F | M | M | M | F | F | M |
| Ferritin | 76 | 2118 | 74 | 428 | 52 | ND | ND | ND | 30 |
| CRP | 3.2 | 6.12 | 10.2 | 1.02 | <0.29 | <0.29 | <0.29 | ND | <0.29 |
| ESR | 65 | ND | 97 | 70 | 6 | 3 | 2 | ND | 4 |
| IL-18 | 15,524 | **60,260** | **17,261** | **63,417** | **6.515** | **3,444** | **2,428** | **1,163** | **8,729** |
| Fever | N | **Y** | **N** | **N** | **N/A** | **N/A** | **N/A** | **N/A** | **N/A** |
| Arthritis | N | **Y** | **Y** | **N** | **N/A** | **N/A** | **N/A** | **N/A** | **N/A** |
| Systemic features | N | **Y** | **N** | **N** | **N/A** | **N/A** | **N/A** | **N/A** | **N/A** |
| Elevated ESR/CRP | Y | **Y** | **Y** | **Y** | **N/A** | **N/A** | **N/A** | **N/A** | **N/A** |
| New-onset SJIA | N | **Y** | **N** | **N** | **N/A** | **N/A** | **N/A** | **N/A** | **N/A** |
| Time in CID | **N/A** | **N/A** | **N/A** | **N/A** | 6mo | 30mo | 48mo | 6mo | 8mo |
| History of MAS | N | Y | N | Y | Y | Y | N | N | Y |
| History of chronic lung disease | N | N | N | Y | N | N | N | N | N |

Supplemental Table 2: DEGs, Active vs control

| Symbol | avg-Active | avg-Control | log_fold-Active_vs_Control | fold-Active_vs_Control | rawp-Active_vs_Control |
| --- | --- | --- | --- | --- | --- |
| EMR1 | 7.87778 | 4.292771 | 3.58501 | 12.00039 | 0.004762 |
| CD274 | 6.449061 | 3.217187 | 3.231875 | 9.394879 | 0.000718 |
| GPR84 | 3.936758 | 1.116286 | 2.820472 | 7.063933 | 0.000388 |
| STOM | 7.551351 | 4.952349 | 2.599002 | 6.058674 | 0.001967 |
| IL6ST | 3.863499 | 1.272089 | 2.59141 | 6.026874 | 0.004214 |
| KLF9 | 3.053132 | 0.54683 | 2.506303 | 5.681621 | 0.047091 |
| TANK | 5.724421 | 3.228947 | 2.495475 | 5.639138 | 0.030111 |
| TNFAIP6 | 8.474419 | 6.095432 | 2.378987 | 5.201714 | 0.00154 |
| FOLR3 | 6.393827 | 4.033082 | 2.360744 | 5.136352 | 0.006924 |
| SLAMF7 | 4.381669 | 2.044851 | 2.336817 | 5.05187 | 0.013387 |
| ANKRD22 | 5.067017 | 2.741429 | 2.325588 | 5.012701 | 0.017099 |
| SERPINB9 | 5.595919 | 3.316406 | 2.279514 | 4.855142 | 0.014669 |
| POU2F2 | 5.07595 | 2.801957 | 2.273993 | 4.8366 | 0.001975 |
| TBC1D8 | 4.088243 | 1.880332 | 2.207911 | 4.62006 | 0.018509 |
| FCGR1A | 7.284552 | 5.096782 | 2.18777 | 4.556006 | 0.019294 |
| CD48 | 6.121926 | 3.974927 | 2.146999 | 4.429056 | 0.000479 |
| TNFAIP3 | 8.24742 | 6.109436 | 2.137983 | 4.401464 | 0.003673 |
| VILL | 3.174553 | 1.038036 | 2.136517 | 4.396993 | 0.012527 |
| TLR5 | 5.546567 | 3.417291 | 2.129277 | 4.374981 | 0.014711 |
| VNN1 | 6.976084 | 4.864284 | 2.1118 | 4.322302 | 0.006808 |
| BATF | 5.689592 | 3.590932 | 2.098661 | 4.283116 | 0.001497 |
| SAMSN1 | 6.352706 | 4.261 | 2.091706 | 4.262518 | 0.02875 |
| AIM2 | 5.347414 | 3.264142 | 2.083272 | 4.237672 | 0.003477 |
| ARID5B | 2.900626 | 0.889104 | 2.011521 | 4.032071 | 0.011914 |
| PLSCR1 | 6.908411 | 5.071618 | 1.836792 | 3.572149 | 0.004685 |
| SMAD3 | 2.93675 | 1.170902 | 1.765847 | 3.400737 | 0.01538 |
| DMXL1 | 3.259313 | 1.497546 | 1.761768 | 3.391134 | 0.044589 |
| NBN | 7.295439 | 5.535262 | 1.760176 | 3.387396 | 0.041724 |
| CLEC4D | 6.197192 | 4.44163 | 1.755562 | 3.376578 | 0.00505 |
| IL18RAP | 7.484018 | 5.746927 | 1.737091 | 3.333623 | 0.006321 |
| THBS1 | 1.730759 | 0 | 1.730759 | 3.319023 | 0.031005 |
| FCGR1C | 3.995418 | 2.268236 | 1.727182 | 3.310804 | 0.026855 |
| GABRR2 | 3.51065 | 1.791414 | 1.719236 | 3.292621 | 0.024923 |
| SLED1 | 8.129483 | 6.455857 | 1.673626 | 3.190154 | 0.0045 |
| P2RY14 | 2.880285 | 1.212263 | 1.668022 | 3.177786 | 0.024083 |
| MOB3C | 5.174458 | 3.522428 | 1.652029 | 3.142754 | 0.006013 |
| GBP3 | 3.16185 | 1.526957 | 1.634893 | 3.105646 | 0.038159 |
| LYZ | 10.37567 | 8.747261 | 1.628412 | 3.091724 | 0.018936 |
| DIAPH2 | 2.683045 | 1.061405 | 1.62164 | 3.077246 | 0.023558 |
| RBM26 | 4.810936 | 3.209466 | 1.601471 | 3.034525 | 0.048569 |
| KLHL5 | 5.337384 | 3.742555 | 1.594828 | 3.020586 | 0.009574 |
| SPTSSA | 2.928211 | 1.340665 | 1.587546 | 3.005377 | 0.010076 |
| RIN3 | 6.455176 | 4.880175 | 1.575001 | 2.979357 | 0.024631 |
| EOGT | 1.979418 | 0.413596 | 1.565822 | 2.96046 | 0.001435 |
| LMNB1 | 3.507939 | 1.945295 | 1.562643 | 2.953946 | 0.005777 |
| ATP6V1C1 | 6.371344 | 4.81224 | 1.559103 | 2.946707 | 0.014346 |
| TIFA | 6.164963 | 4.617921 | 1.547042 | 2.922174 | 0.00244 |
| GRINA | 8.260068 | 6.714809 | 1.545259 | 2.918564 | 0.009146 |
| VRK2 | 4.486356 | 2.948223 | 1.538133 | 2.904184 | 0.027799 |
| ANXA3 | 7.470538 | 5.943967 | 1.526571 | 2.881003 | 0.001808 |
| PLGRKT | 3.866164 | 2.357231 | 1.508933 | 2.845995 | 0.001676 |
| GCH1 | 2.973158 | 1.472605 | 1.500553 | 2.829511 | 0.017284 |
| IL10RA | 4.849942 | 3.36129 | 1.488652 | 2.806266 | 0.006446 |
| MS4A6A | 6.373343 | 4.896737 | 1.476606 | 2.782934 | 0.013193 |
| NME8 | 4.243189 | 2.770499 | 1.47269 | 2.775388 | 0.035482 |
| CTSH | 3.504154 | 2.032787 | 1.471367 | 2.772846 | 0.036661 |
| CR1 | 7.352286 | 5.886978 | 1.465308 | 2.761225 | 0.036472 |
| FCER1G | 10.15259 | 8.699623 | 1.452965 | 2.737702 | 0.046331 |
| RGPD2 | 4.116964 | 2.675167 | 1.441797 | 2.716589 | 0.043069 |
| DDX60L | 8.970352 | 7.533824 | 1.436528 | 2.706687 | 0.007062 |
| SYCP2 | 2.038822 | 0.606294 | 1.432528 | 2.699193 | 0.004738 |
| WDR18 | 1.580907 | 0.156488 | 1.42442 | 2.684065 | 0.001052 |
| NLRC4 | 5.812743 | 4.389829 | 1.422915 | 2.681267 | 0.01985 |
| RSPH9 | 3.372951 | 1.973535 | 1.399416 | 2.637948 | 0.02953 |
| ENTPD7 | 2.331884 | 0.943244 | 1.38864 | 2.618318 | 0.020748 |
| NBR2 | 2.925078 | 1.548238 | 1.37684 | 2.596989 | 0.048312 |
| IFIH1 | 5.672154 | 4.299052 | 1.373102 | 2.590269 | 0.040082 |
| MUSTN1 | 3.653257 | 2.283423 | 1.369834 | 2.584408 | 0.005084 |
| ACOT9 | 6.058188 | 4.690606 | 1.367582 | 2.580377 | 0.010034 |
| FCGR1B | 5.919475 | 4.555284 | 1.364191 | 2.57432 | 0.010665 |
| COPG2 | 1.78038 | 0.417272 | 1.363109 | 2.572389 | 0.000299 |
| EDN1 | 2.601623 | 1.2437 | 1.357923 | 2.563159 | 0.02139 |
| F5 | 5.933723 | 4.576682 | 1.357041 | 2.561592 | 0.006955 |
| GADD45A | 4.026476 | 2.676687 | 1.349789 | 2.548748 | 0.016065 |
| BCKDHA | 3.11211 | 1.769759 | 1.342351 | 2.535642 | 0.003753 |
| TARS | 2.846392 | 1.505741 | 1.340651 | 2.532656 | 0.028765 |
| MEF2A | 5.164699 | 3.836578 | 1.32812 | 2.510754 | 0.019728 |
| LNPEP | 1.789948 | 0.463733 | 1.326216 | 2.507441 | 0.020505 |
| TRIM22 | 9.046262 | 7.721514 | 1.324748 | 2.50489 | 0.005972 |
| ANXA1 | 7.349574 | 6.034791 | 1.314783 | 2.487649 | 0.006487 |
| TLR2 | 8.258208 | 6.947419 | 1.310789 | 2.480772 | 0.047551 |
| CLEC5A | 1.651003 | 0.344021 | 1.306982 | 2.474234 | 0.008155 |
| C11orf82 | 2.660869 | 1.366858 | 1.294011 | 2.452088 | 0.024909 |
| NFKBIA | 10.40283 | 9.109002 | 1.29383 | 2.451781 | 0.006804 |
| IFI16 | 9.483121 | 8.191709 | 1.291412 | 2.447675 | 0.001107 |
| LZTR1 | 1.869178 | 0.578959 | 1.290219 | 2.445652 | 0.033909 |
| MRPS28 | 2.205119 | 0.920412 | 1.284707 | 2.436326 | 0.001333 |
| RHBDF2 | 6.14269 | 4.86332 | 1.27937 | 2.427329 | 0.006908 |
| CYBB | 7.094254 | 5.843155 | 1.251099 | 2.380227 | 0.026243 |
| RNF213 | 9.090371 | 7.842643 | 1.247729 | 2.374673 | 0.037783 |
| MIOS | 2.025934 | 0.782188 | 1.243746 | 2.368127 | 0.040547 |
| ADM | 9.591415 | 8.349776 | 1.241639 | 2.36467 | 0.009631 |
| SLC25A35 | 1.479074 | 0.247968 | 1.231106 | 2.347469 | 0.003892 |
| ORM2 | 5.672833 | 4.463394 | 1.209439 | 2.312476 | 0.04507 |
| OPLAH | 3.467867 | 2.270374 | 1.197493 | 2.293408 | 0.01746 |
| CLEC4E | 9.761471 | 8.56457 | 1.196901 | 2.292467 | 0.026681 |
| OLFM4 | 3.654398 | 2.458687 | 1.195711 | 2.290577 | 0.019374 |
| CD44 | 2.650438 | 1.459476 | 1.190962 | 2.28305 | 0.030259 |
| IRAK2 | 3.505597 | 2.320399 | 1.185198 | 2.273946 | 0.0355 |
| C5 | 1.556537 | 0.37626 | 1.180277 | 2.266202 | 0.01942 |
| MSL3 | 7.603588 | 6.426058 | 1.17753 | 2.261891 | 0.019216 |
| SELP | 2.451098 | 1.275769 | 1.175328 | 2.258443 | 0.040344 |
| LACTB | 3.869842 | 2.702832 | 1.167011 | 2.245459 | 0.049382 |
| ASRGL1 | 1.551521 | 0.386622 | 1.164899 | 2.242175 | 0.007551 |
| GAS7 | 8.697726 | 7.532911 | 1.164815 | 2.242045 | 0.03268 |
| ARHGEF3 | 3.146182 | 1.985433 | 1.160749 | 2.235735 | 0.002419 |
| DDX58 | 8.42634 | 7.266687 | 1.159653 | 2.234036 | 0.015991 |
| FCGR2A | 1.340436 | 0.183627 | 1.156808 | 2.229636 | 0.021307 |
| CAPG | 5.635676 | 4.483394 | 1.152282 | 2.222652 | 0.043922 |
| LOC100129269 | 1.62916 | 0.4789 | 1.15026 | 2.219538 | 0.014384 |
| IGLL3P | 2.287186 | 1.139018 | 1.148169 | 2.216324 | 0.024779 |
| GOLPH3L | 3.443587 | 2.296334 | 1.147253 | 2.214917 | 0.047314 |
| CCDC15 | 2.162304 | 1.024307 | 1.137997 | 2.200752 | 0.009603 |
| CX3CR1 | 4.00665 | 2.868867 | 1.137783 | 2.200426 | 0.007846 |
| INSL3 | 2.639186 | 1.511508 | 1.127678 | 2.185068 | 0.042832 |
| SP100 | 7.628027 | 6.502317 | 1.125709 | 2.182088 | 0.018377 |
| PARP9 | 7.245544 | 6.121397 | 1.124146 | 2.179725 | 0.033263 |
| NRN1 | 1.591355 | 0.471474 | 1.119881 | 2.173291 | 0.032834 |
| RIOK1 | 1.978966 | 0.861272 | 1.117695 | 2.17 | 0.03799 |
| CSNK2A2 | 4.08219 | 2.966474 | 1.115716 | 2.167025 | 0.027874 |
| COQ3 | 1.733832 | 0.621876 | 1.111956 | 2.161385 | 0.01006 |
| KCNJ2 | 6.872502 | 5.763758 | 1.108744 | 2.156578 | 0.012899 |
| FTSJD2 | 6.012628 | 4.907446 | 1.105182 | 2.15126 | 0.047395 |
| CARD17 | 2.246358 | 1.145894 | 1.100464 | 2.144236 | 0.047025 |
| GNA15 | 3.803979 | 2.718654 | 1.085325 | 2.121853 | 0.014246 |
| GK5 | 1.316259 | 0.244641 | 1.071618 | 2.101789 | 0.016049 |
| SLC25A13 | 1.754165 | 0.683992 | 1.070174 | 2.099686 | 0.049303 |
| RNF19A | 2.788392 | 1.726853 | 1.061539 | 2.087157 | 0.018675 |
| PDE1B | 3.263344 | 2.204183 | 1.059162 | 2.08372 | 0.018171 |
| EMC2 | 3.940695 | 2.881859 | 1.058836 | 2.08325 | 0.031592 |
| LGALS8 | 5.135502 | 4.092005 | 1.043497 | 2.061218 | 0.04343 |
| ACSL1 | 11.40261 | 10.36026 | 1.042356 | 2.059589 | 0.017962 |
| SDHAP2 | 5.057266 | 4.016662 | 1.040605 | 2.057089 | 0.009182 |
| FCAR | 6.888207 | 5.848855 | 1.039352 | 2.055304 | 0.042449 |
| DUSP3 | 3.463636 | 2.429408 | 1.034228 | 2.048017 | 0.040092 |
| RGPD1 | 4.739416 | 3.715075 | 1.024342 | 2.034031 | 0.029563 |
| WSB1 | 6.156534 | 5.139015 | 1.017519 | 2.024435 | 0.02621 |
| CNTRL | 4.247198 | 3.23397 | 1.013228 | 2.018422 | 0.011988 |
| NHLH1 | 1.33722 | 0.33064 | 1.00658 | 2.009143 | 0.049354 |
| CCND2 | 1.939904 | 2.952216 | -1.01231 | -2.01714 | 0.011506 |
| NTMT1 | 0.906576 | 1.922323 | -1.01575 | -2.02195 | 0.009646 |
| C1orf85 | 2.639283 | 3.658031 | -1.01875 | -2.02616 | 0.016874 |
| FOXN3-AS1 | 1.009687 | 2.033725 | -1.02404 | -2.0336 | 0.04591 |
| HIC2 | 0.498903 | 1.523268 | -1.02436 | -2.03406 | 0.007817 |
| ZNF559 | 1.659607 | 2.688364 | -1.02876 | -2.04027 | 0.024469 |
| MSH2 | 0.647369 | 1.679525 | -1.03216 | -2.04508 | 0.037244 |
| CXCL2 | 0.322901 | 1.356309 | -1.03341 | -2.04685 | 0.020548 |
| ZNF324 | 3.768765 | 4.809653 | -1.04089 | -2.05749 | 0.015798 |
| RHOF | 3.267414 | 4.30848 | -1.04107 | -2.05775 | 0.013571 |
| ACSS1 | 1.175408 | 2.218783 | -1.04338 | -2.06104 | 0.025489 |
| CTNNBIP1 | 2.706187 | 3.751788 | -1.0456 | -2.06423 | 0.015592 |
| SNORA47 | 4.522699 | 5.574524 | -1.05182 | -2.07315 | 0.035086 |
| TF | 0 | 1.065573 | -1.06557 | -2.093 | 0.028394 |
| TIGD3 | 1.245192 | 2.31682 | -1.07163 | -2.1018 | 0.045751 |
| MIRLET7BHG | 1.462149 | 2.535694 | -1.07354 | -2.1046 | 0.017787 |
| STAP1 | 0 | 1.076153 | -1.07615 | -2.10841 | 0.032245 |
| LCT | 0 | 1.077794 | -1.07779 | -2.11081 | 0.026896 |
| PI4K2A | 0.811416 | 1.897121 | -1.08571 | -2.12241 | 0.028263 |
| TMEM194A | 0.643273 | 1.750734 | -1.10746 | -2.15466 | 0.022412 |
| RASL11A | 1.084706 | 2.194524 | -1.10982 | -2.15818 | 0.007043 |
| PROC | 0.791433 | 1.903061 | -1.11163 | -2.16089 | 0.045425 |
| ASB6 | 2.403273 | 3.519584 | -1.11631 | -2.16792 | 0.040924 |
| ZNF582 | 0.086491 | 1.20473 | -1.11824 | -2.17082 | 0.001002 |
| SNAI3 | 2.423766 | 3.549189 | -1.12542 | -2.18166 | 0.004089 |
| FAM220A | 2.149797 | 3.278862 | -1.12907 | -2.18717 | 0.044026 |
| FAM160A1 | 0 | 1.129449 | -1.12945 | -2.18775 | 0.033672 |
| ELK3 | 2.517578 | 3.665189 | -1.14761 | -2.21547 | 0.038843 |
| FAM134B | 1.274024 | 2.422162 | -1.14814 | -2.21628 | 0.046558 |
| SH3RF3-AS1 | 3.449228 | 4.605549 | -1.15632 | -2.22888 | 0.049526 |
| SNX4 | 1.336337 | 2.511576 | -1.17524 | -2.2583 | 0.02977 |
| HSPB6 | 0 | 1.17763 | -1.17763 | -2.26205 | 0.046093 |
| IRF2BP1 | 3.115151 | 4.300851 | -1.1857 | -2.27474 | 0.031583 |
| KCNK7 | 2.367389 | 3.567213 | -1.19982 | -2.29712 | 0.006136 |
| TCTN1 | 2.064285 | 3.265973 | -1.20169 | -2.30009 | 0.026807 |
| HSPA7 | 4.618535 | 5.845969 | -1.22743 | -2.3415 | 0.017401 |
| MTPAP | 0.597797 | 1.826241 | -1.22844 | -2.34314 | 0.005212 |
| LETM2 | 3.263839 | 4.505973 | -1.24213 | -2.36548 | 0.029804 |
| CBX8 | 1.583243 | 2.82655 | -1.24331 | -2.3674 | 0.014482 |
| PTP4A3 | 4.131365 | 5.375249 | -1.24388 | -2.36835 | 0.025902 |
| LEPREL2 | 0.101226 | 1.354234 | -1.25301 | -2.38338 | 0.022677 |
| TMEM134 | 1.303075 | 2.578003 | -1.27493 | -2.41987 | 0.047836 |
| TMPRSS2 | 0.243355 | 1.53247 | -1.28911 | -2.44378 | 0.02286 |
| RRN3P1 | 0.393423 | 1.699578 | -1.30616 | -2.47282 | 0.001342 |
| SLC7A10 | 0.344858 | 1.662578 | -1.31772 | -2.49272 | 0.024362 |
| LZTFL1 | 2.355709 | 3.698035 | -1.34233 | -2.5356 | 0.0231 |
| AGAP6 | 3.343458 | 4.702561 | -1.3591 | -2.56526 | 0.010566 |
| REPIN1 | 1.359161 | 2.728021 | -1.36886 | -2.58266 | 0.015596 |
| ZNF718 | 0.659638 | 2.030649 | -1.37101 | -2.58652 | 0.006071 |
| SIK1 | 2.234774 | 3.631455 | -1.39668 | -2.63295 | 0.044861 |
| ACP5 | 1.041424 | 2.446695 | -1.40527 | -2.64868 | 0.012066 |
| CRIM1 | 0.244732 | 1.653209 | -1.40848 | -2.65457 | 0.015889 |
| TTC9 | 3.382315 | 4.792209 | -1.40989 | -2.65718 | 0.024615 |
| RERE | 1.836413 | 3.252021 | -1.41561 | -2.66772 | 0.014303 |
| BTBD11 | 0.543099 | 1.976381 | -1.43328 | -2.7006 | 0.029668 |
| CSF1 | 3.94964 | 5.391816 | -1.44218 | -2.7173 | 0.010033 |
| KDM2B | 0.299466 | 1.770924 | -1.47146 | -2.77302 | 0.007152 |
| FAM117B | 1.535593 | 3.041703 | -1.50611 | -2.84043 | 6.22E-05 |
| PPFIBP2 | 1.988972 | 3.517664 | -1.52869 | -2.88524 | 0.036658 |
| SLAMF6 | 0.14213 | 1.681105 | -1.53898 | -2.90588 | 0.001927 |
| ADAM28 | 0.870287 | 2.417531 | -1.54724 | -2.92258 | 0.030532 |
| PMAIP1 | 4.657777 | 6.214337 | -1.55656 | -2.94152 | 0.019908 |
| GNG7 | 3.448265 | 5.036477 | -1.58821 | -3.00677 | 0.017501 |
| HVCN1 | 3.633427 | 5.243998 | -1.61057 | -3.05373 | 0.046757 |
| FAIM3 | 2.689908 | 4.302713 | -1.61281 | -3.05846 | 0.03856 |
| MPRIP | 3.84243 | 5.473868 | -1.63144 | -3.09822 | 0.005395 |
| USP10 | 1.531274 | 3.184823 | -1.65355 | -3.14607 | 0.007524 |
| RUNX3 | 2.320985 | 3.980266 | -1.65928 | -3.15859 | 0.023237 |
| SCCPDH | 1.144444 | 2.806586 | -1.66214 | -3.16486 | 0.013566 |
| CHI3L1 | 7.098873 | 8.818981 | -1.72011 | -3.29461 | 0.009783 |
| CST3 | 6.296034 | 8.028479 | -1.73245 | -3.32291 | 0.006296 |
| CLEC9A | 2.605694 | 4.38078 | -1.77509 | -3.42259 | 0.037012 |
| C19orf60 | 1.892847 | 3.707743 | -1.8149 | -3.51834 | 0.000936 |
| ENC1 | 1.396673 | 3.271732 | -1.87506 | -3.66817 | 0.04696 |
| SDC2 | 1.88307 | 3.772794 | -1.88972 | -3.70564 | 0.030211 |

Supplemental Table 3: Differentially expressed genes between all patients and controls (fold change >2.0, p<0.05).

| Symbol | avg-Patient | avg-Control | log_fold-Patient_vs_Control | fold-Patient_vs_Control | rawp-Patient_vs_Control |
| --- | --- | --- | --- | --- | --- |
| EMR1 | 6.556329 | 4.292771 | 2.263558 | 4.801742 | 0.028084 |
| CD274 | 5.286784 | 3.217187 | 2.069597 | 4.197695 | 0.007703 |
| IL6ST | 3.333356 | 1.272089 | 2.061267 | 4.173526 | 0.001558 |
| GPR84 | 3.076334 | 1.116286 | 1.960047 | 3.890747 | 0.001852 |
| TANK | 4.98479 | 3.228947 | 1.755844 | 3.377238 | 0.042624 |
| CD48 | 5.636749 | 3.974927 | 1.661822 | 3.164158 | 0.000212 |
| POU2F2 | 4.446763 | 2.801957 | 1.644807 | 3.127059 | 0.002704 |
| CLEC4D | 6.057091 | 4.44163 | 1.615461 | 3.064095 | 0.0014 |
| SLAMF7 | 3.620866 | 2.044851 | 1.576014 | 2.981451 | 0.03089 |
| PLSCR1 | 6.593045 | 5.071618 | 1.521426 | 2.870748 | 0.003283 |
| THBS1 | 1.511614 | 0 | 1.511614 | 2.851289 | 0.022527 |
| ANKRD22 | 4.250402 | 2.741429 | 1.508973 | 2.846073 | 0.043165 |
| AIM2 | 4.771178 | 3.264142 | 1.507036 | 2.842256 | 0.010647 |
| RBM26 | 4.700434 | 3.209466 | 1.490968 | 2.810775 | 0.007928 |
| SLED1 | 7.944209 | 6.455857 | 1.488353 | 2.805684 | 0.000381 |
| FCGR1A | 6.566765 | 5.096782 | 1.469983 | 2.770186 | 0.037094 |
| NIPA2 | 4.263055 | 2.800094 | 1.462961 | 2.756735 | 0.022639 |
| NBR2 | 3.002702 | 1.548238 | 1.454464 | 2.740548 | 0.003965 |
| LILRA6 | 9.692427 | 8.251784 | 1.440643 | 2.714418 | 0.003492 |
| EOGT | 1.824444 | 0.413596 | 1.410847 | 2.658933 | 0.000257 |
| CR1 | 7.295397 | 5.886978 | 1.408418 | 2.65446 | 0.004122 |
| ATF2 | 5.550951 | 4.20008 | 1.350872 | 2.550662 | 0.023495 |
| UPB1 | 2.734357 | 1.386374 | 1.347983 | 2.54556 | 0.013452 |
| DMXL1 | 2.844937 | 1.497546 | 1.347391 | 2.544516 | 0.025773 |
| LZTR1 | 1.924403 | 0.578959 | 1.345443 | 2.541083 | 0.007617 |
| TNFAIP6 | 7.436619 | 6.095432 | 1.341187 | 2.533596 | 0.047191 |
| PPAPDC1B | 2.322355 | 0.99549 | 1.326865 | 2.50857 | 0.006194 |
| ITGB1 | 3.548544 | 2.230032 | 1.318512 | 2.494087 | 0.012827 |
| SCP2 | 2.590619 | 1.275854 | 1.314765 | 2.487618 | 0.004859 |
| SMG8 | 3.49376 | 2.19059 | 1.30317 | 2.467705 | 0.019225 |
| NBN | 6.828116 | 5.535262 | 1.292853 | 2.450121 | 0.043086 |
| SS18 | 3.366518 | 2.074551 | 1.291967 | 2.448617 | 0.013706 |
| TLR2 | 8.217164 | 6.947419 | 1.269745 | 2.411189 | 0.00488 |
| F5 | 5.845608 | 4.576682 | 1.268926 | 2.409821 | 0.001874 |
| RIN3 | 6.142224 | 4.880175 | 1.26205 | 2.398362 | 0.02703 |
| P2RY14 | 2.472392 | 1.212263 | 1.260129 | 2.395171 | 0.045452 |
| RBM34 | 3.042796 | 1.782938 | 1.259858 | 2.394722 | 0.012302 |
| SRGAP2B | 3.839786 | 2.582378 | 1.257407 | 2.390657 | 0.011879 |
| COPG2 | 1.674377 | 0.417272 | 1.257105 | 2.390157 | 1.33E-05 |
| ANKDD1A | 2.759075 | 1.505589 | 1.253486 | 2.384168 | 0.006032 |
| TNFAIP3 | 7.330498 | 6.109436 | 1.221061 | 2.331182 | 0.042497 |
| KLHL5 | 4.961245 | 3.742555 | 1.21869 | 2.327353 | 0.013104 |
| XRCC4 | 4.921322 | 3.707436 | 1.213886 | 2.319616 | 0.027629 |
| GCH1 | 2.667073 | 1.472605 | 1.194468 | 2.288604 | 0.042159 |
| ACSL5 | 3.606335 | 2.418162 | 1.188173 | 2.27864 | 0.014761 |
| S100A8 | 11.97061 | 10.78461 | 1.185995 | 2.275202 | 0.01181 |
| ACAD8 | 3.615878 | 2.431519 | 1.184359 | 2.272625 | 0.008005 |
| ATP6V1C1 | 5.996321 | 4.81224 | 1.184081 | 2.272186 | 0.009704 |
| PPP3CB | 3.582676 | 2.398668 | 1.184008 | 2.272071 | 0.03247 |
| BATF | 4.772075 | 3.590932 | 1.181144 | 2.267565 | 0.030963 |
| C14orf101 | 4.249261 | 3.068564 | 1.180697 | 2.266862 | 0.015562 |
| VPS54 | 3.760169 | 2.602565 | 1.157604 | 2.230866 | 0.018658 |
| FCER1G | 9.853201 | 8.699623 | 1.153578 | 2.22465 | 0.025652 |
| RNGTT | 2.649723 | 1.497852 | 1.151872 | 2.22202 | 0.011867 |
| ANXA3 | 7.09098 | 5.943967 | 1.147013 | 2.214549 | 0.001443 |
| ARIH1 | 5.463432 | 4.320526 | 1.142906 | 2.208254 | 0.009064 |
| CCDC90B | 2.840351 | 1.703649 | 1.136702 | 2.198778 | 0.01993 |
| LYZ | 9.880166 | 8.747261 | 1.132906 | 2.193 | 0.034117 |
| COG6 | 1.698487 | 0.566719 | 1.131768 | 2.191271 | 0.018268 |
| VRK2 | 4.071023 | 2.948223 | 1.1228 | 2.177692 | 0.044007 |
| PDE1B | 3.322599 | 2.204183 | 1.118416 | 2.171085 | 0.002118 |
| CDH23 | 3.545901 | 2.429225 | 1.116676 | 2.168468 | 0.010065 |
| RIOK1 | 1.974244 | 0.861272 | 1.112972 | 2.162908 | 0.006722 |
| STK3 | 4.67638 | 3.566355 | 1.110025 | 2.158494 | 0.046841 |
| LOC100129269 | 1.588896 | 0.4789 | 1.109996 | 2.158451 | 0.001358 |
| IL10RA | 4.466808 | 3.36129 | 1.105518 | 2.151761 | 0.006792 |
| CASP1 | 7.494486 | 6.390394 | 1.104093 | 2.149636 | 0.021449 |
| TRAF3IP2 | 4.149282 | 3.055683 | 1.093598 | 2.134057 | 0.035562 |
| FCGR1B | 5.647008 | 4.555284 | 1.091724 | 2.131286 | 0.008875 |
| PLGRKT | 3.445702 | 2.357231 | 1.088471 | 2.126486 | 0.003046 |
| STARD3NL | 5.485593 | 4.404669 | 1.080924 | 2.115391 | 0.029877 |
| SLC44A1 | 2.987617 | 1.919349 | 1.068268 | 2.096914 | 0.036996 |
| SETDB2 | 2.574639 | 1.507657 | 1.066981 | 2.095045 | 0.014525 |
| COG4 | 4.398279 | 3.340265 | 1.058014 | 2.082063 | 0.007061 |
| EMC2 | 3.934213 | 2.881859 | 1.052354 | 2.07391 | 0.003384 |
| ALG6 | 3.316653 | 2.264386 | 1.052267 | 2.073786 | 0.031002 |
| NRN1 | 1.522828 | 0.471474 | 1.051354 | 2.072475 | 0.021499 |
| VPS9D1 | 4.95776 | 3.909683 | 1.048076 | 2.067771 | 0.044221 |
| ELMO1 | 6.673889 | 5.627023 | 1.046866 | 2.066036 | 0.007328 |
| CYBB | 6.880361 | 5.843155 | 1.037206 | 2.05225 | 0.033568 |
| CLEC4E | 9.597454 | 8.56457 | 1.032884 | 2.04611 | 0.00829 |
| SDHAP2 | 5.044558 | 4.016662 | 1.027897 | 2.03905 | 0.000576 |
| CLEC5A | 1.365557 | 0.344021 | 1.021535 | 2.030078 | 0.016752 |
| IPO8 | 5.340532 | 4.319657 | 1.020875 | 2.02915 | 0.009037 |
| OPLAH | 3.289206 | 2.270374 | 1.018832 | 2.026278 | 0.013021 |
| ACOT9 | 5.708901 | 4.690606 | 1.018295 | 2.025523 | 0.010192 |
| TAF8 | 3.834299 | 2.81626 | 1.018039 | 2.025165 | 0.003584 |
| CSNK2A2 | 3.983894 | 2.966474 | 1.01742 | 2.024296 | 0.003095 |
| PPP2R4 | 3.294785 | 2.278347 | 1.016438 | 2.022919 | 0.012307 |
| TRMT1L | 4.683943 | 3.66997 | 1.013973 | 2.019465 | 0.024079 |
| ENTPD7 | 1.957138 | 0.943244 | 1.013894 | 2.019354 | 0.014131 |
| TIFA | 5.629215 | 4.617921 | 1.011295 | 2.015719 | 0.015651 |
| OPA1 | 1.798079 | 0.787101 | 1.010978 | 2.015276 | 0.01207 |
| SLK | 6.552542 | 5.542907 | 1.009635 | 2.013402 | 0.023657 |
| THADA | 2.662518 | 1.657603 | 1.004915 | 2.006825 | 0.008478 |
| NCOR1 | 4.884264 | 3.881476 | 1.002787 | 2.003868 | 0.006659 |
| RNF19A | 2.726955 | 1.726853 | 1.000102 | 2.000142 | 0.002081 |
| LCT | 0.070532 | 1.077794 | -1.00726 | -2.01009 | 0.002424 |
| ESRP2 | 1.87334 | 2.880758 | -1.00742 | -2.01031 | 0.024307 |
| BIN1 | 0.096873 | 1.109678 | -1.0128 | -2.01783 | 0.027466 |
| DEFA5 | 0.389232 | 1.410041 | -1.02081 | -2.02906 | 0.023355 |
| PCYT2 | 0.632233 | 1.653822 | -1.02159 | -2.03015 | 0.025019 |
| PMAIP1 | 5.192731 | 6.214337 | -1.02161 | -2.03018 | 0.037905 |
| TUBB2A | 0.37243 | 1.409346 | -1.03692 | -2.05184 | 0.013859 |
| GADD45G | 2.721387 | 3.760769 | -1.03938 | -2.05535 | 0.038092 |
| KRT25 | 0.49598 | 1.540381 | -1.0444 | -2.06251 | 0.031797 |
| SH3RF3-AS1 | 3.558023 | 4.605549 | -1.04753 | -2.06698 | 0.011139 |
| LOC399815 | 0.540358 | 1.591991 | -1.05163 | -2.07287 | 0.010252 |
| ZGLP1 | 2.456967 | 3.512936 | -1.05597 | -2.07912 | 0.042555 |
| CST3 | 6.971133 | 8.028479 | -1.05735 | -2.0811 | 0.033826 |
| CREBZF | 4.885353 | 5.946799 | -1.06145 | -2.08702 | 0.026106 |
| HDGFL1 | 0.260234 | 1.32391 | -1.06368 | -2.09025 | 0.037948 |
| TTC9 | 3.721661 | 4.792209 | -1.07055 | -2.10023 | 0.027022 |
| THAP11 | 3.768937 | 4.839624 | -1.07069 | -2.10043 | 0.011006 |
| SNORA36A | 3.659799 | 4.7313 | -1.0715 | -2.10162 | 0.029897 |
| ACP5 | 1.373099 | 2.446695 | -1.0736 | -2.10467 | 0.025675 |
| RNF113A | 3.871982 | 4.951878 | -1.0799 | -2.11388 | 0.005956 |
| SNORA70B | 4.981234 | 6.063714 | -1.08248 | -2.11767 | 0.028987 |
| TMPRSS2 | 0.449176 | 1.53247 | -1.08329 | -2.11887 | 0.013782 |
| FAM132A | 2.819508 | 3.907741 | -1.08823 | -2.12614 | 0.047143 |
| RGS3 | 0.452428 | 1.549534 | -1.09711 | -2.13925 | 0.04083 |
| C1orf85 | 2.543708 | 3.658031 | -1.11432 | -2.16493 | 0.000387 |
| LOC400657 | 1.789705 | 2.909104 | -1.1194 | -2.17256 | 0.018797 |
| GABPB1-AS1 | 2.910269 | 4.030011 | -1.11974 | -2.17308 | 0.026228 |
| KDM2B | 0.641126 | 1.770924 | -1.1298 | -2.18828 | 0.009943 |
| ELK3 | 2.529934 | 3.665189 | -1.13526 | -2.19657 | 0.027755 |
| HSPB6 | 0.041439 | 1.17763 | -1.13619 | -2.198 | 0.004803 |
| SLC7A10 | 0.519756 | 1.662578 | -1.14282 | -2.20812 | 0.007608 |
| LOC642361 | 3.291889 | 4.437474 | -1.14558 | -2.21236 | 0.01393 |
| FLJ42289 | 0.597575 | 1.749042 | -1.15147 | -2.2214 | 0.046569 |
| C3orf65 | 0.518522 | 1.671111 | -1.15259 | -2.22313 | 0.025938 |
| SUV39H1 | 2.692651 | 3.847422 | -1.15477 | -2.22649 | 0.040993 |
| APC2 | 1.028057 | 2.198104 | -1.17005 | -2.25019 | 0.030892 |
| ATP1B4 | 0.391422 | 1.56539 | -1.17397 | -2.25631 | 0.048806 |
| CXCL6 | 3.22228 | 4.402426 | -1.18015 | -2.266 | 0.048423 |
| INGX | 2.311041 | 3.49489 | -1.18385 | -2.27182 | 0.040952 |
| TERC | 3.252861 | 4.438919 | -1.18606 | -2.2753 | 0.042518 |
| CKMT2 | 0.21045 | 1.408827 | -1.19838 | -2.29481 | 0.027023 |
| RBMX2 | 2.595852 | 3.806806 | -1.21095 | -2.31491 | 0.030026 |
| FLJ23867 | 4.538947 | 5.755681 | -1.21673 | -2.3242 | 0.014636 |
| RPL13 | 3.216587 | 4.449952 | -1.23337 | -2.35115 | 0.02422 |
| CSNK2A1 | 4.585416 | 5.835532 | -1.25012 | -2.37861 | 0.03557 |
| KRT86 | 0.486705 | 1.737304 | -1.2506 | -2.3794 | 0.029331 |
| PRODH2 | 0.581649 | 1.837483 | -1.25583 | -2.38805 | 0.033956 |
| EGR3 | 3.745625 | 5.021667 | -1.27604 | -2.42174 | 0.022333 |
| EGR2 | 2.137774 | 3.417127 | -1.27935 | -2.4273 | 0.02678 |
| BLOC1S4 | 2.576085 | 3.861942 | -1.28586 | -2.43827 | 0.039393 |
| KRT73 | 1.238594 | 2.525424 | -1.28683 | -2.43991 | 0.049478 |
| TPBGL | 0.598128 | 1.892564 | -1.29444 | -2.45281 | 0.020885 |
| CNPY2 | 2.233124 | 3.549752 | -1.31663 | -2.49083 | 0.048522 |
| ZCCHC16 | 2.92222 | 4.255746 | -1.33353 | -2.52018 | 0.021558 |
| SSBP4 | 2.183031 | 3.545445 | -1.36241 | -2.57115 | 0.039055 |
| DPPA4 | 0.354434 | 1.773867 | -1.41943 | -2.6748 | 0.029069 |
| PRTN3 | 0.85749 | 2.277524 | -1.42003 | -2.67592 | 0.02979 |
| LOC641367 | 3.023884 | 4.475811 | -1.45193 | -2.73573 | 0.034813 |
| EBPL | 4.02399 | 5.492126 | -1.46814 | -2.76664 | 0.028962 |
| ZNF718 | 0.536469 | 2.030649 | -1.49418 | -2.81704 | 0.000437 |
| TNP2 | 0.366836 | 1.873252 | -1.50642 | -2.84103 | 0.011838 |
| LOC440461 | 2.241805 | 3.849193 | -1.60739 | -3.047 | 0.032465 |
| UPK3B | 3.961585 | 5.602317 | -1.64073 | -3.11824 | 0.04736 |
| PPIAL4C | 0.844238 | 2.486476 | -1.64224 | -3.1215 | 0.040276 |
| C9orf152 | 1.241001 | 2.888297 | -1.6473 | -3.13246 | 0.038436 |
| RCN1 | 3.104233 | 4.761158 | -1.65693 | -3.15344 | 0.045935 |
| USP26 | 1.476667 | 3.176581 | -1.69991 | -3.24882 | 0.041396 |
| NKAPL | 3.777928 | 5.486031 | -1.7081 | -3.26731 | 0.025003 |
| THOC3 | 2.213341 | 3.93065 | -1.71731 | -3.28822 | 0.0458 |
| AARD | 2.084829 | 3.876204 | -1.79137 | -3.46145 | 0.032255 |
| C5orf55 | 2.14535 | 4.022484 | -1.87713 | -3.67345 | 0.049673 |
| KRTAP26-1 | 1.638576 | 3.536087 | -1.89751 | -3.7257 | 0.043239 |
| GCSH | 2.269792 | 4.172759 | -1.90297 | -3.73981 | 0.039728 |
| GNG7 | 3.130804 | 5.036477 | -1.90567 | -3.74684 | 0.011134 |
| LSP1P3 | 1.792423 | 3.790625 | -1.9982 | -3.99502 | 0.035491 |
| LOC392364 | 2.006024 | 4.007073 | -2.00105 | -4.00291 | 0.045956 |
| FOXD4L1 | 1.918879 | 4.069571 | -2.15069 | -4.44041 | 0.047193 |
| TREML5P | 3.756179 | 5.911631 | -2.15545 | -4.45508 | 0.037731 |
| MIR548I3 | 1.679621 | 3.872875 | -2.19325 | -4.57336 | 0.037582 |
| OR6Y1 | 1.701608 | 4.037427 | -2.33582 | -5.04837 | 0.026111 |
| CETN1 | 1.885909 | 4.383169 | -2.49726 | -5.64612 | 0.025691 |

Supplemental table 4:

|  | 1 | 2 | 3 | 4 | 5 | 6 | 7 | 8 | 9 | 10 |
| --- | --- | --- | --- | --- | --- | --- | --- | --- | --- | --- |
| Disease activity | CID | CID | CID | CID | CID | CID | CID | CID | CID | CID |
| Age | 7 | 11 | 5 | 16 | 1 | 21 | 7 | 9 | 6 | 13 |
| Sex | M | F | M | M | F | F | F | M | M | F |
| Ferritin | ND | ND | 33 | ND | 21 | ND | ND | 26 | 15 | 21 |
| CRP | ND | <0.29 | <0.29 | <0.29 | <0.29 | <0.29 | <0.29 | <0.29 | <0.29 | <0.29 |
| ESR | ND | 3 | 3 | 4 | 7 | 4 | 5 | 11 | 5 | 16 |
| IL-18 | 1,188 | **164** | **4,247** | **587** | **387** | **<31** | **335** | **626** | **281** | **1,695** |
| Fever | **N/A** | **N/A** | **N/A** | **N/A** | **N/A** | **N/A** | **N/A** | **N/A** | **N/A** | **N/A** |
| Arthritis | **N/A** | **N/A** | **N/A** | **N/A** | **N/A** | **N/A** | **N/A** | **N/A** | **N/A** | **N/A** |
| Systemic features | **N/A** | **N/A** | **N/A** | **N/A** | **N/A** | **N/A** | **N/A** | **N/A** | **N/A** | **N/A** |
| Elevated ESR/CRP | **N/A** | **N/A** | **N/A** | **N/A** | **N/A** | **N/A** | **N/A** | **N/A** | **N/A** | **N/A** |
| New-onset SJIA | **N/A** | **N/A** | **N/A** | **N/A** | **N/A** | **N/A** | **N/A** | **N/A** | **N/A** | **N/A** |
| Time in CID | **30mo** | 18mo | 6mo | 36mo | 12mo | 6mo | 14mo | 20mo | 18mo | 1mo |
| History of MAS | N | N | Y | N | N | N | N | N | N | N |
| History of chronic lung disease | N | N | N | N | N | N | N | N | N | N |
